# Global Budget Revenue Model and Care for Patients Receiving Chemotherapy

**DOI:** 10.1001/jamanetworkopen.2026.0485

**Published:** 2026-03-05

**Authors:** Yu-Li Lin, Bradley Herring, Alexander Melamed, Laura A. Petrillo, Nancy L. Keating, Anaeze C. Offodile

**Affiliations:** 1 Department of Health Services Research, University of Texas MD Anderson Cancer Center, Houston; 2 Department of Economics, University of New Hampshire Paul College of Business and Economics, Durham; 3 Department of Health Management and Policy, University of New Hampshire College of Health and Human Services, Durham; 4 Department of Obstetrics and Gynecology, Massachusetts General Hospital, Boston; 5 Division of Palliative Care and Geriatrics, Massachusetts General Hospital, Boston; 6 Department of Health Care Policy, Harvard Medical School, Boston, Massachusetts; 7 Division of General Internal Medicine, Brigham and Women’s Hospital, Boston, Massachusetts; 8 Plastic and Reconstructive Surgery Service, Memorial Sloan Kettering Cancer Center, New York, New York

## Abstract

**Importance:**

Maryland’s Global Budget Revenue (GBR) model provided fixed global payments to hospitals, aiming to control revenue growth while improving care quality. The implications of GBR implementation in 2014 for cancer care have not been robustly evaluated.

**Objective:**

To examine the association between GBR implementation and subsequent changes in episode-based Medicare payments, hospital utilization, and quality of care among Medicare beneficiaries undergoing systemic therapy for cancer.

**Design, Setting, and Participants:**

This cohort study used a difference-in-differences approach to compare changes in outcome measures from pre-GBR (2011-2013) to post-GBR (2014-2018) implementation periods in Maryland compared with 11 control states. Six-month care episodes were selected for adult fee-for-service Medicare beneficiaries initiating or continuing cytotoxic chemotherapy, immunotherapy, or targeted therapy for cancer between 2011 and 2018. Data were obtained from Medicare claims, including inpatient, outpatient, carrier, durable medical equipment, home health agency, hospice, and Part D event files. All analyses were performed between April 4, 2024, and January 5, 2026.

**Main Outcomes and Measures:**

The primary outcomes were standardized Medicare payments during the 6-month systemic therapy episode, including total, hospital, and professional payments. Also assessed were measures of hospital-based utilization (all-cause hospitalizations and emergency department [ED] visits) and care quality (timely receipt of chemotherapy; chemotherapy-related hospitalizations and ED visits; and measures of high-intensity end-of-life treatment: no or late hospice enrollment, >1 ED visit in the last 30 days of life, intensive care unit stay in the last 30 days of life, and receipt of chemotherapy in the last 14 days of life).

**Results:**

A total of 38 531 chemotherapy episodes in Maryland were matched to 38 531 episodes in control states. Episodes in Maryland were for patients (22 185 females [57.6%]) with a mean (SD) age of 73.3 (8.6) years; in control states, episodes were for patients (21 708 females [56.3%]) with a mean (SD) age of 72.7 (9.1) years. GBR implementation was associated with a reduction of $3075 (95% CI, −$4276 to −$1843; 6.1% savings) in total episode payments, a reduction of $3217 (95% CI, −$4058 to −$2328; 17.3% savings) in hospital payments, and an increase of $1382 (95% CI, $781-$2013; 11.9% increase) in professional payments. There was a reduction of 1.7 (95% CI, −3.0 to −0.5) percentage points in chemotherapy-related hospitalizations. No significant association was found for other hospital-based utilization or care quality measures.

**Conclusions and Relevance:**

This cohort study of patients with systemic anticancer therapy episodes showed that Maryland’s GBR model was associated with substantial reductions in the growth of Medicare payments. These savings may have been achieved by shifting care toward lower-cost treatment settings.

## Key Points

**Question:**

How did Maryland’s Global Budget Revenue (GBR) model affect the payments, hospital utilization, and quality of care for Medicare beneficiaries undergoing systemic therapy for cancer?

**Findings:**

In this cohort study of 77 062 total chemotherapy episodes, the GBR model’s implementation was associated with a relative reduction in total Medicare payments, hospital-based payments, and chemotherapy-related hospitalizations in Maryland compared with control states, along with a larger increase in professional payments during 6 months of systemic therapy episodes. Measures of quality of care did not change.

**Meaning:**

The findings of this study suggest that Maryland’s GBR model achieved substantial reductions in the growth of Medicare payments for patients undergoing systemic therapy for cancer, possibly by shifting care toward lower-cost treatment settings.

## Teaser

This cohort study examines changes in Medicare payments, hospital utilization, and care quality for anticancer therapy after the 2014 implementation of the Global Budget Revenue program in Maryland.

## Introduction

In 2025, there were 2 041 910 incident cancer cases and 618 120 cancer-related deaths reported in the US.^1^ The costs of cancer care are consequential, estimated at more than $180 billion in 2015 with a projected increase to $246 billion by 2030.^2^ Medicare bears a disproportionate share of these costs, as the median age at cancer diagnosis in the US is 67 years.^3^ In recent decades, policymakers have identified alternative payment models (APMs) as a potential strategy to address cancer’s unsustainable spending trajectory.^4,5,6^

Historically, Maryland administered an all-payer rate-setting system, which resulted in greater per-beneficiary Medicare spending for chronic conditions than in most other states.^7^ In January 2014, Maryland and the Centers for Medicare & Medicaid Services (CMS) implemented a statewide Global Budget Revenue (GBR) model that provided hospital-specific, prospectively set total revenue caps across all sites of care and payers (commercial insurance, Medicare, and Medicaid). The GBR program’s primary objective was to control the total spending growth for hospital services in Maryland (<3.6% per annum) while incentivizing high-quality care delivery.^8^ The implementation of GBR was associated with nearly $1 billion in savings to the Medicare program, the highest^9^ among all APMs to date, without demonstrable deleterious implications for care quality.^10^

However, GBR’s program evaluations have not robustly explored its role in specialty care, despite specialty care accounting for 95% of Medicare’s payments.^11,12^ With planned expansions of the global budget model to other states,^13^ considering the effectiveness in cancer care of such reforms is a policy priority. More than 10% of US adult nonmaternal hospital stays are cancer related,^14^ and acute hospital care is a key factor in overall spending and spending variation among Medicare beneficiaries with advanced cancer.^15^ Moreover, anticancer drugs—often administered in hospital outpatient departments—comprise the largest portion of spending for patients with cancer undergoing systemic therapy.^5^ Hospitals exposed to GBR are thus incentivized to control cancer-related utilization to meet the annual revenue cap. Therefore, we expected GBR to be associated with a slowing in the growth of Medicare payments. In the present study, we used a difference-in-differences (DID)^16^ framework to examine the association between GBR implementation and subsequent changes in episode-based Medicare payments, hospital utilization, and quality of care among Medicare beneficiaries undergoing systemic therapy for cancer.

## Methods

### Study Design

Our DID approach characterized the preimplementation to postimplementation changes in the treated group (patient care episodes in Maryland) compared with the control states. The DID approach allows a comparison of these changes even if there are different levels in the preintervention period outcomes, as long as there are nondifferential trends during the preintervention period.^16^ The pre-GBR implementation period included 6-month care episodes for patients initiating systemic therapy from January 1, 2011, to June 30, 2013, and the post-GBR implementation period included episodes initiated from January 1, 2014, to June 30, 2018. The institutional review boards of the Memorial Sloan Kettering Cancer Center and the University of Texas MD Anderson Cancer Center deemed this study exempt from ethics review and informed consent requirement because it was secondary research. We followed the Strengthening the Reporting of Observational Studies in Epidemiology (STROBE) reporting guideline.^17^

### Data Sources and Study Population

We used fee-for-service Medicare claims data from January 1, 2010, to December 31, 2018, including Medicare Beneficiary Summary Files; claims for inpatient, outpatient, carrier, durable medical equipment, home health, and hospice services; and Part D event files. Following the methods of the Oncology Care Model (OCM),^18^ we constructed 6-month systemic anticancer therapy episodes for patients initiating or continuing cytotoxic chemotherapy, immunotherapy, or targeted therapy (hereafter, chemotherapy); unlike the OCM, we did not include hormonal therapies. We included Medicare beneficiaries in Maryland and 11 control states: Connecticut, Delaware, Illinois, Massachusetts, Michigan, New Jersey, New York, Ohio, Rhode Island, Vermont, and West Virginia. These control states are in adjacent Census divisions and—similar to Maryland—expanded their Medicaid program in 2014, minimizing potential spillover effects from Medicaid expansion coincident with GBR’s rollout.^19^

Details on identifying the chemotherapy episodes are provided in eAppendix 1 and eTable 1 in Supplement 1. Briefly, we selected episodes for adult patients (aged 18 years or older) with a cancer diagnosis who initiated chemotherapy and were continuously enrolled in Medicare Parts A and B in the prior year and during all months of the episode or until death if within an episode. Each episode was assigned to a treating oncologist and oncology practice based on the plurality of office visits. Episodes attributed to out-of-state oncology practices were excluded. Before GBR’s implementation, rural hospitals in Maryland were exposed to a fixed annual revenue cap via a 3-year pilot program, the Total Patient Revenue (TPR) model.^20^ Therefore, we excluded episodes from practices in Hospital Service Areas (HSAs) served by hospitals enrolling in the TPR model or TPR-eligible hospitals (including similar hospitals in the control states).

Additionally, we constructed 2 subsamples to examine GBR’s implications for the (1) timely initiation of adjuvant chemotherapy after curative-intent surgery and (2) use of appropriate end-of-life care. The first subsample focused on episodes for patients with breast, lung, or colorectal cancer with a curative surgical procedure in the 180 days before chemotherapy initiation (eTable 2 in Supplement 1).^18^ The end-of-life cohort included patients who died during or within 90 days after the end of the episode.

### Dependent Variables

We examined 3 categories of dependent variables: standardized Medicare payments (hereafter, payments, the primary study outcome), hospital-based utilization, and care quality measures. Payments included total episode payments (payments for inpatient hospital, outpatient hospital, carrier, home health, hospice, Medicare Part D, and durable medical equipment claims), total episode hospital payments (ie, payments in the inpatient and outpatient hospital files), and total episode professional payments (ie, payments in the carrier file). Because Medicare payments vary across geographic areas and are notably higher in Maryland due to the state’s all-payer rate and because Maryland administers its global budget by periodically adjusting hospital payments based on changes in observed hospital utilization,^10^ for each service, we standardized hospital payments to be common across all states; we also standardized payments for each nonhospital service for consistency (eAppendix 2 in Supplement 1). We initially planned to examine chemotherapy payments (total and Part B only); however, the parallel trends assumption was not met for these outcomes (eTable 3 in Supplement 1).

Hospital-based utilization included all-cause hospitalizations and all-cause emergency department (ED) visits not resulting in hospitalizations during the episode. For care quality, we followed the OCM evaluation approach to construct 7 care quality measures.^18^ These measures included timely receipt of chemotherapy (within 60 days of curative-intent surgery) for patients with breast, lung, or colorectal cancer; chemotherapy-related hospitalizations and ED visits^21^; and 4 measures of high-intensity end-of-life treatment^22,23^: no or late (within 3 days of death) hospice enrollment, more than 1 ED visit in the last 30 days of life, intensive care unit stay in the last 30 days of life, and receipt of chemotherapy in the last 14 days of life. When identifying all-cause and chemotherapy-related ED visits, we followed CMS quality reporting specifications^21^ and reported the rate of ED visits without hospitalization during the episode. However, for end-of-life treatment, we counted all ED visits in the last 30 days of life.^22,23^

### Control Variables

Patient age, sex, race and ethnicity, dual eligibility status, and continuous Part D enrollment during the episode were obtained from Medicare data. Race and ethnicity (Asian or Pacific Islander, Hispanic, non-Hispanic Black, non-Hispanic White, and other (including American Indian or Alaska Native, other race, and unknown race) were included in this analysis because the cancer care outcome varies by race and ethnicity.^24^ Chemotherapy type (clinic or hospital administered [Part B] or self-administered [Part D]) was determined based on the first chemotherapy claim. Cancer type was assigned using the diagnosis on the first chemotherapy claim. We used all inpatient, outpatient, and carrier claims during the episode to calculate the disability index^25^ and identify metastatic disease. Institutional status was determined by any nursing facility’s Evaluation and Management claims in the outpatient and carrier files in the 90 days before the episode.^26^ We documented chemotherapy episodes and comorbidity using Hierarchical Condition Categories in the preceding year. We characterized the Social Deprivation Index^27^ and the percentage of uninsured persons in patients’ zip code of residence.

We documented year-specific HSA-level variables, including number of billing oncologists per 10 000 elderly population, oncology practice–defined Herfindahl-Hirschman index,^28^ number of hospital-owned oncology practices, total number of beds, percentage of 340B hospitals, and percentage of teaching hospitals. We defined hospital-owned practices as those with 90% or greater of claims in an outpatient hospital setting. Total number of beds and percentage of teaching hospitals were estimated from the 2011 to 2018 American Hospital Association Annual Survey. The percentage of hospitals with 340B eligibility was estimated from Health Resources and Services Administration data.^29^

### Matching

To identify comparable chemotherapy episodes across Maryland and control states, we performed a 2-step matching process. First, we matched Maryland HSAs to HSAs in control states based on degree of urbanization in 2013 and time trends in hospital and professional standardized payments during the pre-GBR implementation period (2011 to 2013). Second, within each set of matched HSAs, we matched chemotherapy episodes by treatment year and patient characteristics using 1:1 propensity score matching. eAppendix 3 in Supplement 1 includes additional details.

### Statistical Analysis

We compared absolute standardized differences in episode characteristics for Maryland and control states before and after the 2-step matching. For each outcome measure, we estimated the annual adjusted mean in Maryland and control states, adjusting for all patient and HSA-level characteristics. For payments, we fitted a γ distribution with the log link function. For the other binary outcome measures (hospital-based utilization and quality of care), we used a linear probability model. The unit of analysis was the chemotherapy episode, except for the end-of-life analyses, for which the unit was the patient. Patients could have more than 1 episode; robust SEs were used to account for patient clustering. Details on the statistical modeling are provided in eAppendix 4 in Supplement 1.

We used a DID approach to examine changes over time in Maryland’s outcome compared with the control states. Our main model included the year of implementation (2014) in the postimplementation period. Sensitivity analyses excluded the impact from the year of implementation, allowing a 12-month washout period, assuming that GBR-related practice changes may take time.^30^ This exclusion also reduced the risk of carryover bias during the immediate postpolicy period, when any observable treatment outcomes are less likely to reflect true policy impact.^30^ In secondary analyses, we used an event study DID approach to assess if the impact of GBR varied over time after implementation.

All DID models were adjusted for patient covariates, time-varying HSA-level characteristics, and HSA fixed effects. Details of the model specification for DID estimation and testing the parallel trends assumption are provided in eAppendix 5 in Supplement 1. Two-sided *P* < .05 were considered statistically significant. We presented false discovery rate–adjusted *P* values, in addition to original *P* values, to account for testing multiple outcomes. All analyses were performed between April 4, 2024, and January 5, 2026, using SAS Enterprise Guide, version 7.15 (SAS Institute Inc).

## Results

### Matching Episodes

After matching, 38 531 chemotherapy episodes in Maryland were matched to 38 531 episodes in control states. Matched episodes in Maryland were for patients with a mean (SD) age of 73.3 (8.6) years and included 22 185 episodes (57.6%) for females and 16 346 (42.4%) for males. In control states, the included episodes were for patients with a mean (SD) age of 72.7 (9.1) years and included 21 708 episodes (56.3%) for females and 16 823 (43.7%) for males (Table 1). Before matching, 460 episodes (1.2%) in Maryland and 1341 (0.2%) in control states that were missing payments or matching variables were excluded. After matching, the parallel trends assumption for the DID analysis was not violated for each measure (eTable 3 in Supplement 1).

**Table 1.** Patient and Hospital Service Area Characteristics for Chemotherapy Episodes Before and After Matching in Maryland and Control States

Characteristic	Chemotherapy episodes, No. (%)					
	Before matching			After matching^a^		
	Maryland (n = 38 541)	Control states (n = 640 564)	ASD	Maryland (n = 38 531)	Control states (n = 38 531)	ASD
**Patients**						
Age at chemotherapy episode initiation, mean (SD), y	73.3 (8.6)	73.2 (9.0)	0.02	73.3 (8.6)	72.7 (9.1)	0.08
Frailty index, mean (SD)^b^	0.2 (0.1)	0.2 (0.1)	0.07	0.2 (0.1)	0.2 (0.1)	0.00
SDI of zip code, mean (SD)^c^	40.3 (28.9)	41.6 (29.4)	0.04	40.3 (28.9)	40.4 (29.7)	0.00
% Of uninsured people in zip code, mean (SD)	7.7 (5.2)	7.7 (5.6)	0.00	7.7 (5.2)	7.9 (5.4)	0.03
Sex						
Male	16 351 (42.4)	283 832 (44.3)	0.04	16 346 (42.4)	16 823 (43.7)	0.03
Female	22 190 (57.6)	356 732 (55.7)		22 185 (57.6)	21 708 (56.3)	
Race and ethnicity^d^						
Asian or Pacific islander	1146 (3.0)	11 584 (1.8)	0.46	1146 (3.0)	1264 (3.3)	0.08
Hispanic	783 (2.0)	23 968 (3.7)		783 (2.0)	763 (2.0)	
Non-Hispanic Black	9621 (25.0)	58 651 (9.2)		9611 (24.9)	9129 (23.7)	
Non-Hispanic White	26 397 (68.5)	534 390 (83.4)		26 397 (68.5)	26 762 (69.5)	
Other^e^	594 (1.5)	11 971 (1.9)		594 (1.5)	613 (1.6)	
Dual eligibility during episode	5551 (14.4)	116 756 (18.2)	0.10	5551 (14.4)	5751 (14.9)	0.01
Continuous Part D enrollment during episode	25 264 (65.6)	478 879 (74.8)	0.20	25 262 (65.6)	24 945 (64.7)	0.02
Initial chemotherapy of episode						
Part B	32 509 (84.3)	521 483 (81.4)	0.08	32 501 (84.4)	32 501 (84.4)	0.00
Part D	6032 (15.7)	119 081 (18.6)		6030 (15.6)	6030 (15.6)	
Cancer type						
Acute leukemia	513 (1.3)	7296 (1.1)	0.22	513 (1.3)	521 (1.4)	0.08
Bladder cancer	1182 (3.1)	19 295 (3.0)		1182 (3.1)	1190 (3.1)	
Breast cancer	5406 (14.0)	79 883 (12.5)		5406 (14.0)	5393 (14.0)	
Chronic leukemia	1857 (4.8)	34 944 (5.5)		1857 (4.8)	1875 (4.9)	
Endocrine tumor	630 (1.6)	10 971 (1.7)		630 (1.6)	608 (1.6)	
Female genitourinary cancer	828 (2.1)	14 350 (2.2)		828 (2.1)	816 (2.1)	
Gastro or esophageal cancer	945 (2.5)	16 771 (2.6)		945 (2.5)	1029 (2.7)	
Head and neck cancer	739 (1.9)	13 747 (2.1)		739 (1.9)	717 (1.9)	
Kidney cancer	398 (1.0)	7929 (1.2)		398 (1.0)	418 (1.1)	
Liver cancer	441 (1.1)	8946 (1.4)		441 (1.1)	432 (1.1)	
Lung cancer	6338 (16.4)	101 936 (15.9)		6337 (16.4)	6301 (16.4)	
Lymphoma	4218 (10.9)	68 229 (10.7)		4218 (10.9)	4218 (10.9)	
Myelodysplastic syndromes	964 (2.5)	15 950 (2.5)		964 (2.5)	981 (2.5)	
Multiple myeloma	4318 (11.2)	55 708 (8.7)		4312 (11.2)	4297 (11.2)	
Ovarian cancer	1357 (3.5)	22 368 (3.5)		1357 (3.5)	1354 (3.5)	
Pancreatic cancer	1415 (3.7)	22 603 (3.5)		1414 (3.7)	1330 (3.5)	
Prostate cancer	1032 (2.7)	18 613 (2.9)		1031 (2.7)	1036 (2.7)	
Metastasis to other and unspecified sites	486 (1.3)	16 110 (2.5)		486 (1.3)	541 (1.4)	
Metastasis to respiratory and digestive organs	222 (0.6)	10 584 (1.7)		222 (0.6)	239 (0.6)	
Colorectal and small intestine cancer	2779 (7.2)	51 461 (8.0)		2778 (7.2)	2790 (7.2)	
Other	2473 (6.4)	42 870 (6.7)		2473 (6.4)	2445 (6.3)	
Metastasis during episode						
Distant	15 690 (40.7)	289 751 (45.2)	0.11	15 689 (40.7)	15 525 (40.3)	0.02
Lymph node	1294 (3.4)	24 565 (3.8)		1293 (3.4)	1291 (3.4)	
None	21 557 (55.9)	326 248 (50.9)		21 549 (55.9)	21 715 (56.4)	
Any nursing facility service in prior 90 d	1475 (3.8)	30 176 (4.7)	0.04	1475 (3.8)	1547 (4.0)	0.01
Chemotherapy episode in the prior 12 mos	20 941 (54.3)	350 724 (54.8)	0.01	20 938 (54.3)	20 591 (53.4)	0.02
Diabetes						
No diabetes	25 006 (64.9)	416 825 (65.1)	0.03	25 002 (64.9)	25 206 (65.4)	0.00
Diabetes without complication	6940 (18.0)	119 507 (18.7)		6938 (18.0)	6958 (18.1)	
Diabetes with complication	6595 (17.1)	104 232 (16.3)		6591 (17.1)	6367 (16.5)	
**HSA **						
No. of billing oncologist per 10 000 elderly population, mean (SD)	11.3 (4.2)	12.5 (12.6)	0.13	11.3 (4.2)	10.3 (10.0)	0.14
Competition: oncology practice-defined HHI, mean (SD)^f^	0.4 (0.2)	0.5 (0.3)	0.49	0.4 (0.2)	0.5 (0.3)	0.56
No. of hospital-owned oncology practices, mean (SD)	0.5 (0.5)	2.3 (3.8)	0.66	0.5 (0.5)	1.6 (2.4)	0.65
Total No. of beds, mean (SD)	3891.7 (2666.7)	1979.9 (2600.3)	0.73	3892.6 (2666.4)	1705.8 (1851.6)	0.95
% Of 340B hospitals, mean (SD)	57.7 (40.9)	69.2 (56.4)	0.23	57.7 (40.8)	62.1 (48.0)	0.10
% Of teaching hospitals, mean (SD)	67.2 (34.3)	66.4 (39.4)	0.02	67.2 (34.3)	68.8 (34.5)	0.05

Abbreviations: ASD, absolute standardized difference; HHI, Herfindahl-Hirschman index; HSA, hospital service area; SDI, Social Deprivation Index.

^a^ A 2-step matching procedure was used to match Maryland’s episodes to episodes from the control states. The first step matched Maryland HSAs to a subset of control state HSAs based on urbanization and utilization trends, and the second step matched episodes (within matched HSAs) based on patient characteristics. HSA characteristics were not included in either step of the matching procedure.

^b^ Frailty index ranges from 0 to 1, with higher values indicating greater frailty.

^c^ SDI ranges from 0-100, with higher values indicating greater neighborhood-level disadvantage.

^d^ Race and ethnicity were initially based on self-reports and then refined using Centers for Medicare & Medicaid Services–developed algorithms.

^e^ Before matching, in Maryland the other category included 0.01% of American Indian or Alaska Native, 0.70% of other race, and 0.83% of unknown race. In control states, it included 0.07% of American Indian or Alaska Native, 0.86% of other race, and 0.94% of unknown race.

^f^ HHI ranges from 0 to 1, with higher values indicating less competition.

Table 1 and eTable 4 in eAppendix 6 in Supplement 1 describe patient-level and HSA-level characteristics before and after matching for the primary sample. Before matching, race and ethnicity, dual eligibility, continuous Part D enrollment during episode, cancer type, and metastasis during episode were unbalanced, with absolute standardized differences greater than 0.1. After matching, all patient-level characteristics were balanced. Unbalanced HSA-level characteristics for hospitals and clinicians were not included in the propensity score matching because we included only episodes from control HSAs with similar payment trends. Similarly, we achieved balance in patient characteristics after matching for the study populations to assess timely receipt of adjuvant chemotherapy and end-of-life care (eTables 5 and 6 in Supplement 1).

### DID Results

**Figure 1.**
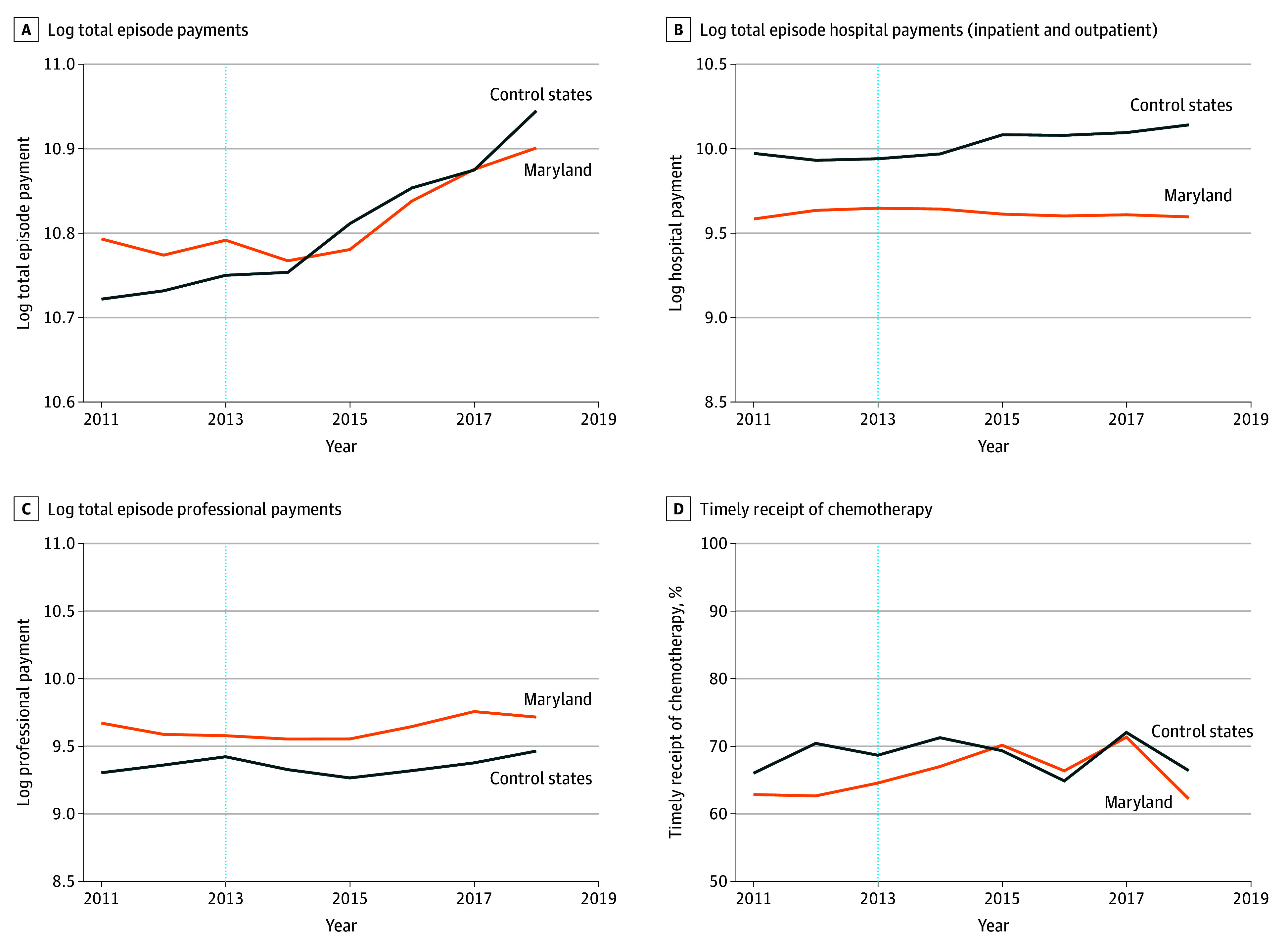
Line Graphs of Adjusted Time Trends for Standardized Medicare Payments and the Timeliness of Chemotherapy All models were adjusted for patient age, sex, race and ethnicity, dual eligibility, institutional status, disability index, Part D enrollment, Part B or D chemotherapy, cancer type, metastasis status, any prior chemotherapy episode, comorbidity using Hierarchical Condition Categories groups, zip code–level Social Deprivation Index and percentage of uninsured people, and year-specific Hospital Service Area–level variables (number of billing oncologists per 10 000 older population, oncology practice–defined Herfindahl-Hirschman index, number of hospital-owned oncology practices, total number of beds, percentage of 340B hospitals, and percentage of teaching hospitals).

**Figure 2.**
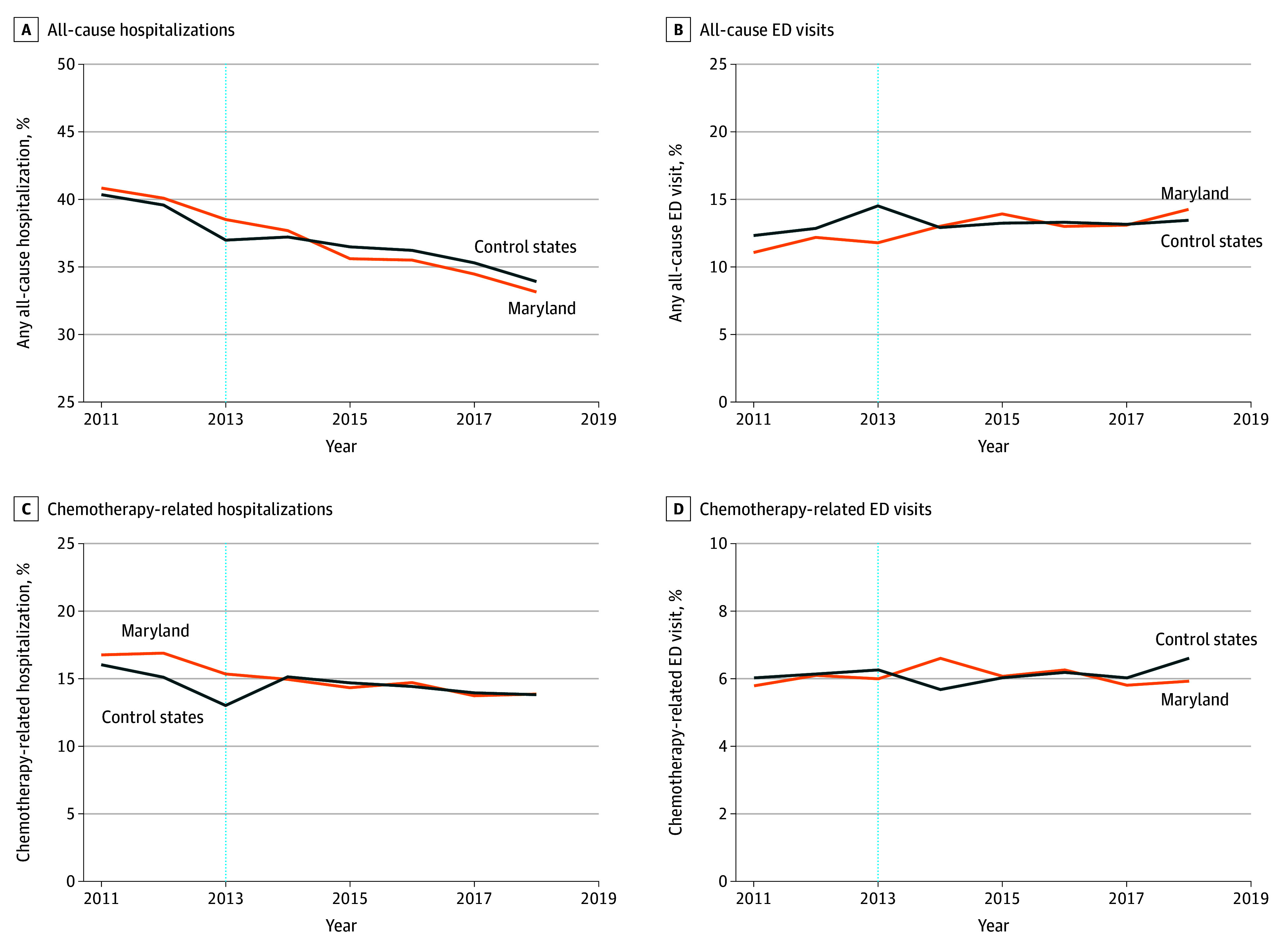
Line Graphs of Adjusted Time Trends for All-Cause and Chemotherapy-Related Hospitalizations and Emergency Department (ED) Visits All models were adjusted for patient age, sex, race and ethnicity, dual eligibility, institutional status, disability index, Part D enrollment, Part B or D chemotherapy, cancer type, metastasis status, any prior chemotherapy episode, comorbidity using Hierarchical Condition Categories groups, zip code–level Social Deprivation Index and percentage of uninsured people, and year-specific Hospital Service Area–level variables (number of billing oncologists per 10 000 older population, oncology practice–defined Herfindahl-Hirschman index, number of hospital-owned oncology practices, total number of beds, percentage of 340B hospitals, and percentage of teaching hospitals).

**Figure 3.**
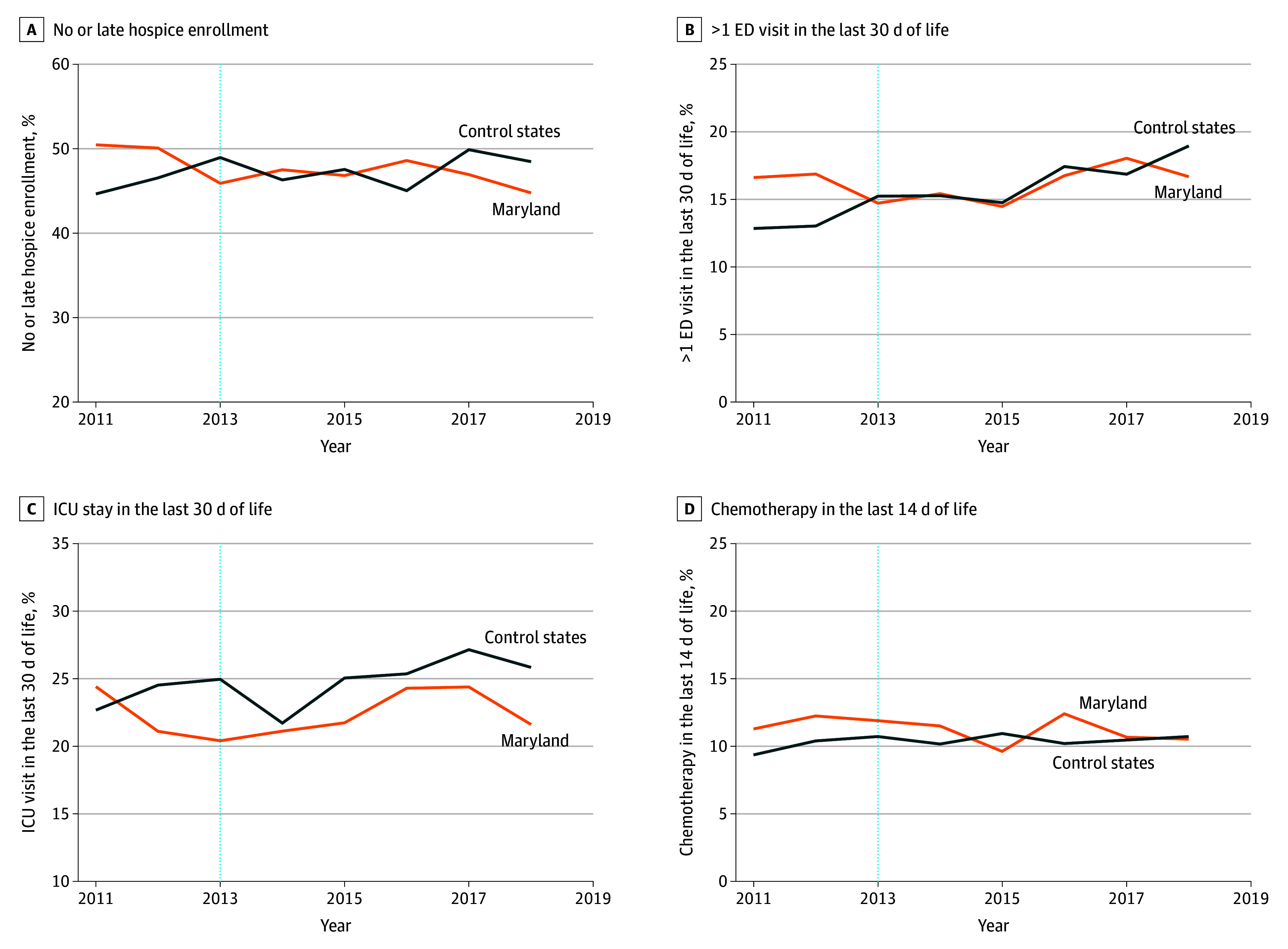
Line Graphs of Adjusted Time Trends for Each Measure of High-Intensity End-of-Life Treatments All models were adjusted for patient age, sex, race and ethnicity, dual eligibility, institutional status, disability index, Part D enrollment, Part B or D chemotherapy, cancer type, metastasis status, any prior chemotherapy episode, comorbidity using Hierarchical Condition Categories groups, zip code–level Social Deprivation Index and percentage of uninsured people, and year-specific Hospital Service Area–level variables (number of billing oncologists per 10 000 elderly population, oncology practice–defined Herfindahl-Hirschman index, number of hospital-owned oncology practices, total number of beds, percentage of 340B hospitals, and percentage of teaching hospitals). ED indicates emergency department, and ICU indicates intensive care unit.

Trends in adjusted means of study outcomes by year in Maryland and control states are shown in Figure 1, Figure 2, and Figure 3. Table 2 presents adjusted means (SDs) in the GBR preimplementation and postimplementation periods for Maryland and control states and the DID estimate. Total episode payments increased less after GBR implementation in Maryland compared with control states (DID, −$3075 [95% CI, −$4276 to −$1843]; 6.1% savings); similarly, there were statistically significant reductions in hospital payments (DID, −$3217 [95% CI, −$4058 to −$2328]; 17.3% savings). In contrast, professional payments increased in Maryland vs control states after GBR implementation (DID, $1382 [95% CI, $781-$2013]; 11.9% increase).

**Table 2.** Adjusted Means for Each Outcome Measure and Difference-in-Differences Estimates

Outcome measure	Adjusted mean (SD), $ or %^a^				DID estimates^a^		
	Pre-GBR implementation (2011-2013)		Post-GBR implementation (2014-2018)		DID (95% CI), $ or percentage points	*P* value	FDR-adjusted *P* value^b^
	Maryland	Control states	Maryland	Control states			
Payments							
Total episode payments	$48 345 ($513)	$45 899 ($659)	$50 700 ($2949)	$51 512 ($3689)	−$3075 (−$4276 to −$1843)	<.001	<.001
Total episode hospital payments (inpatient and outpatient)	$15 123 ($505)	$20 924 ($448)	$14 972 ($275)	$23 748 ($1466)	−$3217 (−$4058 to −$2328)	<.001	<.001
Total episode professional payments	$14 958 ($774)	$11 654 ($692)	$15 494 ($1435)	$11 532 ($878)	$1382 ($781 to $2013)	<.001	<.001
Hospital-based utilization							
All-cause hospitalizations	39.8 (1.2)	39.0 (1.8)	35.3 (1.7)	35.8 (1.3)	−1.0 (−2.5 to 0.5)	.17	.30
All-cause ED visits	11.7 (0.6)	13.2 (1.1)	13.5 (0.6)	13.2 (0.2)	1.3 (0.0 to 2.6)	.04	.11
Quality of care							
Chemotherapy-related hospitalizations	16.3 (0.9)	14.7 (1.5)	14.3 (0.5)	14.4 (0.5)	−1.7 (−3.0 to −0.5)	.007	.02
Chemotherapy-related ED visits	6.0 (0.2)	6.1 (0.1)	6.1 (0.3)	6.1 (0.3)	0.3 (−0.6 to 1.2)	.47	.62
Timely receipt of chemotherapy	63.4 (1.0)	68.4 (2.2)	67.5 (3.6)	68.8 (3.1)	5.1 (−1.9 to 12.1)	.15	.30
No or late hospice enrollment^c^	48.8 (2.5)	46.7 (2.2)	46.9 (1.4)	47.5 (1.9)	−0.1 (−3.9 to 3.7)	.95	.95
>1 ED visit in last 30 d of life	16.1 (1.2)	13.7 (1.3)	16.3 (1.4)	16.7 (1.7)	−1.6 (−4.5 to 1.2)	.26	.39
ICU stay in last 30 d of life	22.0 (2.1)	24.1 (1.2)	22.7 (1.6)	25.1 (2.0)	0.4 (−2.8 to 3.6)	.80	.93
Receipt of chemotherapy in last 14 d of life	11.8 (0.5)	10.1 (0.7)	10.9 (1.1)	10.5 (0.3)	0.2 (−2.2 to 2.7)	.85	.93

Abbreviations: DID, difference-in-differences; ED, emergency department; FDR, false discovery rate; GBR, Global Budget Revenue; ICU, intensive care unit.

^a^ All models were adjusted for patient age, sex, race and ethnicity, dual eligibility, institutional status, disability index, Part D enrollment, Part B or D chemotherapy, cancer type, metastasis status, any prior chemotherapy episode, comorbidity using Hierarchical Condition Categories groups, zip code–level Social Deprivation Index and percentage of uninsured people, and year-specific Hospital Service Area (HSA)–level variables (number of billing oncologists per 10 000 elderly population, oncology practice–defined Herfindahl-Hirschman index, number of hospital-owned oncology practices, total number of beds, percentage of 340B hospitals, and percentage of teaching hospitals). Year (based on chemotherapy initiation) fixed effects were also included. DIDs calculated directly from the adjusted means (presented in the first 4 columns) are slightly different from the DID estimates (presented in the fifth column) because the regression models that generated the adjusted means for the former estimates did not include HSA fixed effects, while the latter estimates included HSA fixed effects.

^b^ This adjustment approach is also known as Benjamini-Hochberg procedure. We investigated 12 outcome variables and applied the Benjamini-Hochberg procedure to adjust these 12 *P* values.

^c^ Late hospice enrollment is defined as hospice enrollment within 3 days of death.

All-cause hospitalizations declined similarly across Maryland and the control states after GBR implementation, and we did not observe a significant DID (−1.0 [95% CI, −2.5 to 0.5] percentage points). In contrast, all-cause ED visits without a hospitalization increased slightly in Maryland compared with control states during the post-GBR implementation period (DID, 1.3 [95% CI, 0.0-2.6] percentage points); this finding lost statistical significance with false discovery rate adjustment and when the year of implementation was excluded from the postimplementation period (eTable 7 in eAppendix 7 in Supplement 1). Chemotherapy-related hospitalizations were higher in Maryland than in control states in the preimplementation period and decreased more than in control states (DID, −1.7 [95% CI, −3.0 to −0.5] percentage points). There were no statistically significant differences in chemotherapy-related ED visits, timeliness of chemotherapy initiation, or measures of aggressive end-of-life care in Maryland compared with control states after GBR implementation. With 26 079 unique episodes in Maryland between 2014 and 2018 in the initial study population, we estimated that the combined total savings attributable to the GBR model for patients undergoing chemotherapy for cancer was $80.2 million.

The event study findings were generally consistent with the DID findings (eFigure in Supplement 1). For most measures with differences after GBR implementation, the differences were relatively stable from 2015 through 2018. An exception was hospital payments, which declined slightly more each year (eFigure in Supplement 1). Except for all-cause ED visits, DID results were similar for the sensitivity analyses that excluded the year of implementation from the postimplementation period (eTable 7 in Supplement 1).

## Discussion

In the present cohort study, the implementation of the GBR model in Maryland was associated with a statistically significant $3075 reduction (6.1% decrease) in per-episode standardized payments for Medicare beneficiaries receiving chemotherapy. This finding was partly attributed to lower payments to hospitals even while professional payments increased, suggesting the savings may have been achieved by a shift in sites of care toward lower-cost treatment settings (ie, from hospital outpatient departments to physician offices). There was a small relative increase in all-cause ED visits as well as a relative reduction in chemotherapy-related hospitalizations. Other measures of hospital-based utilization and quality of care did not change.

In recent years, the potential of population-based APMs to improve specialty care delivery has drawn substantial attention, especially given CMS’s stated goal of transitioning Medicare beneficiaries to value-based care arrangements.^11,31,32^ Cancer is one of the most expensive chronic conditions in the US^2^ and the second leading cause of mortality, justifying its policy salience. An independent evaluation of the OCM—the largest cancer-related APM, with more than 3000 participating oncologists—found modest reductions in Medicare payments (DID, −$616 [95% CI, −$912 to −$321]; 2.1% reduction) for comparable 6-month episodes.^33^ Although reductions in total episode payments increased over time, OCM did not achieve its goal of reducing total Medicare spending because savings did not offset model payments. OCM did not improve or worsen care quality or patient experience. The per-episode payment reductions in our study were substantially greater than those seen in OCM. GBR’s design specifically targeted hospital-based spending. Maryland hospitals may have leveraged site-of-care optimization strategies in response to GBR’s revenue cap, such as an accelerated shift of surgical^19^ or other hospital-based care from hospital inpatient to outpatient settings. Further work is needed to confirm a shift in sites of care for patients undergoing chemotherapy, including understanding what services (eg, chemotherapy, imaging) were shifted to nonhospital settings.

Compared with episode payment models, population-based APMs, such as GBR, may allow organizations to refine and improve the allocation of health services for chronic conditions such as cancer.^31^ They offer health care delivery organizations, such as hospitals, considerable regulatory flexibilities and necessary alignment of incentives to maximize outcomes per unit cost—provided that the appropriate utilization, patient access, and quality measures are prospectively set. While it is encouraging that we found no evidence of worsening quality, our quality measures were primarily measures of overuse of care, which is already disincentivized with GBR. New measures addressing potential underuse of care are needed to ensure that appropriate care is delivered. Extending the locus of accountability to include community practitioners will also be important for achieving durable, system-wide transformation, as up to 80% of US cancer care is delivered in community settings.^34,35^ In a recent qualitative study^36^ examining the perspectives of Maryland health care leaders on the implementation of GBR, the following themes were identified: increased hospital autonomy, use of actionable data for stakeholder engagement, and a shared commitment to change. A similar inquiry at the level of oncologists would be helpful in discerning the contributions of enhanced ambulatory symptom management, care coordination, and advance care planning to our findings of reduced total Medicare payments and increased utilization of professional services rather than hospital services.^5^ Further study should investigate the likelihood of chemotherapy initiation under GBR. In addition, it will be important to evaluate GBR’s implications for care for commercially insured patients. Lastly, it will be essential to ensure that these GBR-related changes in care delivery do not exacerbate existing inequities for marginalized or socioeconomically vulnerable patients with cancer.

### Limitations

This study has limitations. First, we studied care through 2018. In 2019, Maryland transitioned to the Total Cost of Care model, expanding the regulated domains from hospitals only to include primary care physicians and specialists. Second, the specificity of the Maryland all-payer GBR model context limits the generalizability of our findings to states without similar regulatory environments. Third, we leveraged Medicare fee-for-service claims for our analysis, and the findings may not generalize to Medicare Advantage, Medicaid, or commercially insured patients. Fourth, we lacked clinical details, such as disease stage and tumor markers, as well as information about patient preferences on end-of-life care. Although we conducted a rigorous DID analysis with an appropriately matched control group, we cannot exclude the possibility of unmeasured confounding. Moreover, although we used several measures of quality, most of our measures assessed potential overuse of care; the lack of clinical details limited our ability to examine more nuanced measures of quality, including appropriate use of recommended care.

## Conclusions

In this cohort study of patients undergoing chemotherapy, we found that Maryland’s GBR model was associated with substantial reductions in standardized Medicare payment growth. As states consider the broader adoption of population-based APMs such as GBR, consideration should be given to cancer-specific quality safeguards, meaningful engagement of community practitioners, and vigilance for adverse spillover effects. Future research should assess the model’s applicability beyond fee-for-service Medicare beneficiaries.

## Notes

Supplement 1. **eAppendix 1.** Constructing the Initial Cohort of Chemotherapy Episodes **eTable 1.** Initial Cohort Selection **eAppendix 2.** Standardized Payment Methodology **eAppendix 3.** Matching Strategy **eTable 2.** ICD and CPT Codes Used for Identifying Curative Surgeries for Breast, Lung, and Colorectal Cancers **eAppendix 4.** Model for Estimating Adjusted Means **eAppendix 5.** Estimating Difference-in-Differences (DiD) and Testing the Parallel Trends Assumption **eTable 3.** Test for Parallel Trends Assumption **eFigure.** Difference-in-Differences Each Year With the Year 2013 as the Reference **eAppendix 6.** Description of the Matched Samples **eTable 4.** Comorbidity for Chemotherapy Episodes Before and After Matching in Maryland and Control States – Main Sample **eTable 5.** Patient Demographic, Clinical, and Area Characteristics for Chemotherapy Episodes Before and After Matching in Maryland and Control States – Subsample 1 for Timely Receipt of Presumed Adjuvant Chemotherapy for Patients With Breast, Lung, and Colorectal Cancers **eTable 6.** Patient Demographic, Clinical, and Area Characteristics for Chemotherapy Episodes Before and After Matching in Maryland and Control States – Subsample 2 for End-of-Life Measures **eAppendix 7.** Sensitivity Analyses **eTable 7.** Adjusted Means for Each Outcome Measure and Difference-in-Differences Estimates
eReferences

Supplement 2.
Data Sharing Statement
