# Supplementary material for: Global Budget Revenue Model and Care for Patients Receiving Chemotherapy
Source: JAMA Netw Open. 2026 Mar 5;9(3):e260485. doi: 10.1001/jamanetworkopen.2026.0485 (PMC12964157; doi:10.1001/jamanetworkopen.2026.0485)
Supplement: Supplement 1. — eAppendix 1. Constructing the Initial Cohort of Chemotherapy Episodes eTable 1. Initial Cohort Selection eAppendix 2. Standardized Payment Methodology eAppendix 3. Matching Strategy eTable 2. ICD and CPT Codes Used for Identifying Curative Surgeries for Breast, Lung, and Colorectal Cancers eAppendix 4. Model for Estimating Adjusted Means eAppendix 5. Estimating Difference-in-Differences (DiD) and Testing the Parallel Trends Assumption eTable 3. Test for Parallel Trends Assumption eFigure. Difference-in-Differences Each Year With the Year 2013 as the Reference eAppendix 6. Description of the Matched Samples eTable 4. Comorbidity for Chemotherapy Episodes Before and After Matching in Maryland and Control States – Main Sample eTable 5. Patient Demographic, Clinical, and Area Characteristics for Chemotherapy Episodes Before and After Matching in Maryland and Control States – Subsample 1 for Timely Receipt of Presumed Adjuvant Chemotherapy for Patients With Breast, Lung, and Colorectal Cancers eTable 6. Patient Demographic, Clinical, and Area Characteristics for Chemotherapy Episodes Before and After Matching in Maryland and Control States – Subsample 2 for End-of-Life Measures eAppendix 7. Sensitivity Analyses eTable 7. Adjusted Means for Each Outcome Measure and Difference-in-Differences Estimates eReferences [file jamanetwopen-e260485-s001.pdf]

Supplemental Online Content

Lin YL, Herring B, Melamed A, Petrillo LA, Keating NL, Offodile AC. Global budget revenue model and care for patients receiving chemotherapy. *JAMA Netw Open*. 2026;9(3):e260485. doi:10.1001/jamanetworkopen.2026.0485

eAppendix 1. Constructing the Initial Cohort of Chemotherapy Episodes

eTable 1. Initial Cohort Selection

eAppendix 2. Standardized Payment Methodology

eAppendix 3. Matching Strategy

eTable 2. ICD and CPT Codes Used for Identifying Curative Surgeries for Breast, Lung, and Colorectal Cancers

eAppendix 4. Model for Estimating Adjusted Means

eAppendix 5. Estimating Difference-in-Differences (DiD) and Testing the Parallel Trends Assumption

eTable 3. Test for Parallel Trends Assumption

eFigure. Difference-in-Differences Each Year With the Year 2013 as the Reference

eAppendix 6. Description of the Matched Samples

eTable 4. Comorbidity for Chemotherapy Episodes Before and After Matching in Maryland and Control States – Main Sample

eTable 5. Patient Demographic, Clinical, and Area Characteristics for Chemotherapy Episodes Before and After Matching in Maryland and Control States – Subsample 1 for Timely Receipt of Presumed Adjuvant Chemotherapy for Patients With Breast, Lung, and Colorectal Cancers

eTable 6. Patient Demographic, Clinical, and Area Characteristics for Chemotherapy Episodes Before and After Matching in Maryland and Control States – Subsample 2 for End-of-Life Measures

eAppendix 7. Sensitivity Analyses

© 2026 Lin YL et al. *JAMA Network Open*

eTable 7. Adjusted Means for Each Outcome Measure and Difference-in-Differences Estimates

eReferences

This supplemental material has been provided by the authors to give readers additional information about their work.

© 2026 Lin YL et al. *JAMA Network Open*

eAppendix 1. Constructing the Initial Cohort of Chemotherapy Episodes

We followed the methodology from the Oncology Care Model (OCM)[1] to construct 6-month chemotherapy episodes for cancer using Medicare claims in two time periods: pre-GBR (Jan 2011 to June 2013) and post-GBR (Jan 2014 to June 2018). The process of identifying the initial cohort of chemotherapy episodes was described in eTable 1.

In the study cohort (eTable 1), each episode must have had at least 1 E&M visit with an oncologist during the episode. The methodology for identifying medical oncologists was developed by Pragya Kakani.[2] We followed this methodology and used data between 2010 and 2020 to identify oncologists for our study.

© 2026 Lin YL et al. *JAMA Network Open*

eTable 1. Initial Cohort Selection

	Maryland		Control states		
	n	%, prior step	n	%, prior step	n, total
1. Identify chemotherapy episodes triggered‡ in Maryland or any control state† between 1/1/2011 and 6/30/2013 (pre-Global Budget Revenue [GBR]); 1/1/2014 and 6/30/2018 (post-GBR)	72,824		948,481		1,021,305
2. Select chemotherapy episodes for beneficiaries 18 or older at the beginning of the episode	72,824	100.0	948,477	100.0	1,021,301
3. Select chemotherapy episodes for beneficiaries with continuous enrollment in Parts A&B in the prior year and during all months of the episode, or till death if they died within 6 months of the trigger claim.	64,566	88.7	812,884	85.7	877,450
4. Remove episodes for beneficiaries with end-stage renal disease	63,677	98.6	802,200	98.7	865,877
5. Select those with at least 1 E&M visit with an oncologist during the episode. Each episode was assigned a treating oncologist and practice* that had the most E&M visits.	55,506	87.2	706,753	88.1	762,259
6. Exclude episodes cared for by an out-of-state oncology practice.	54,705	98.6	699,274	98.9	753,979
7. Remove episodes from practices in Hospital Service Areas of Maryland's Total Patient Revenue (TPR) Model or TPR-eligible hospitals.	39,903	72.9	665,589	95.2	705,492
8. Remove episodes from practices in counties without any hospitals.	39,903	100.0	664,514	99.8	704,417
9. Remove episodes with claims or Part D Event records missing standard payment estimate.#	39,444	98.8	663,470	99.8	702,914
10. Remove episodes with missing data for matching variables.	39,443	100.0	663,173	100.0	702,616
11. Select episodes in Hospital Service Areas that 1) appear in both pre- (2011-2013) and post-GBR (2014-2018) periods; 2) had 3 years of data in the pre-period; 3) had more than 10 episodes each year in the pre-period.	38,541	97.7	640,564	96.6	679,105

‡Chemotherapy episodes must be triggered in the same state as the patients' residence. †Control states include Connecticut, Delaware, Illinois, Massachusetts, Michigan, New Jersey, New York, Ohio, Rhode Island, Vermont, West Virginia. *Identified by tax identification number. #PDE records lacking generic names would not have an estimated standard payment. For claims in Maryland, if the billing codes did not appear in any of the control states during 2011-2018, they would not have an estimated standard payment amount. ESRD=end-stage renal disease. TPR=Total Patient Revenue.

© 2026 Lin YL et al. *JAMA Network Open*

eAppendix 2. Standardized Payment Methodology

Overall Approach

We standardized payments because Maryland's all-payer rates are much higher than Medicare payments in other states and because Maryland administers its global budget by periodically adjusting the hospital prices based on changes in observed hospital utilization. To obtain standardized payments, we used data from control states (Connecticut, Delaware, Illinois, Massachusetts, Michigan, New Jersey, New York, Ohio, Rhode Island, Vermont, West Virginia) for the entire study period (2011 through 2018). We first used the Medical Care Consumer Price Index (CPI) to adjust the payment amounts in all years to 2018 dollars. Next, we calculated the average payment for each DRG grouping / HCPCS code, per service count, using all data in all control states from 2011 through 2018.

Notes:

1. Denied claims and claims with a non-Medicare primary payer were excluded. We identified claims as denied if the Medicare non-payment reason code was not blank; the allowed charge was not positive; the payment amount was zero. We did not exclude chemotherapy claims with zero payment amounts because Medicare reimburses $0 for low-cost clinician-administered drugs in hospital outpatient departments as part of the Prospective Payment System (e.g., drugs with prices <$130 in 2020).
2. For calculating Part B chemotherapy payments, we combined outpatient, carrier, and Durable Medical Equipment (DME) claims to calculate the standard payment because Medicare pays the same for Part B chemotherapy regardless of whether the claim was provided in a hospital outpatient department or a physician's office.

Inpatient (MedPAR Files)

Medicare uses Diagnosis Related Groups (DRGs), a classification system that groups patients with similar diagnoses, treatments, and resource needs into a single payment rate for hospitals in the Inpatient Prospective Payment System (IPPS). Many DRG conditions or treatments have 2 or 3 DRG categories that each correspond to a different level of severity. Previous findings[3,4] and our own exploratory analysis show that the highest severity DRGs are under-utilized in Maryland. Therefore, we took the average payment of these 2 or 3 DRG categories to calculate the standard payment for a given condition or treatment.

MedPAR files include admissions in acute care hospitals, long term care hospitals, psychiatric facilities, inpatient rehabilitation facilities, skilled nursing home facilities. They also include admissions in IPPS-exempt facilities, such as critical access hospitals, children's hospitals, religious Nonmedical Health Care Institutions, and some cancer hospitals. The payment rates vary by the facility type; therefore, we computed different standard payments based on the type of stays.

Our price-standardization method has a limitation. The payments to inpatient rehabilitation facilities are based on the Case Mix Groups (CMGs) and the payments to skilled nursing home facilities are based on the Resource Utilization Groups (RUGs). However, CMGs are RUGs are not available in MedPAR files. Standardized payments for stays in these facilities and IPPS-exempt facilities were still calculated based on the DRGs on the MedPAR records.

Steps for estimating standard payment:

1. Consolidate DRGs of various levels of severity.
2. Select inpatient claims from facilities in the 11 control states between 2011 and 2018 with Medicare as the primary payer. Remove denied claims (those with zero payment).
3. Categorize the stays to 8 types:

© 2026 Lin YL et al. *JAMA Network Open*

- Critical access hospitals
- Children's hospitals (excluded from PPS)
- Rehabilitation hospitals
- Long-term hospitals
- Psychiatric hospitals
- Religious Nonmedical Health Care Institutions
- Skilled nursing facilities
- Short-term (general and specialty) hospitals

4. Adjust the payment amount to 2018 dollars using the Medical Care CPI.
5. Compute the average payment for each DRG grouping and each type of stay.
6. Prorate the payment based on number of days in the episode if the stay extended outside the chemotherapy episode.

Outpatient and Carrier

Steps for estimating standard payment:

1. Select claims from providers in the 11 control states between 2011 and 2018. Select claims with Medicare as the primary payer. Remove denied claims and claims with zero payment.
2. Adjust the payment amount to 2018 dollars using the Medical Care CPI.
3. Compute the average payment per service unit for each HCPCS/CPT code.

Durable Medical Equipment

Steps for estimating standard payment:

1. Select claims from providers in the 11 control states between 2011 and 2018. Select claims with Medicare as the primary payer. Remove denied claims and claims with zero payment.
2. Adjust the payment amount to 2018 dollars using the Medical Care CPI.
3. Compute the average payment per service unit for each HCPCS/CPT code.

As per the *CMS Standardization Methodology For Allowed Amount – v.13 (updated August 2024),*[5] payments for some DME line items should be adjusted as indicated by HCPCS code modifiers (see Table 2. Adjustment Factors for DME in the *CMS Standardization Methodology*).

Home Health Agency

As per the *CMS Standardization Methodology For Allowed Amount – v.13 (updated August 2024),*[5] there are 4 types of Home Health Agency (HHA) episodes:

- full HHA episode/period
- partial episode/period payment (PEP)
- low utilization payment adjustment (LUPA) episode/period
- a request for anticipated payment (RAP)

For the full and PEP episodes, the payment depends on the Home Health Resource Groups (HHRG) and the duration (in days) of HHA episodes. An approach to calculate the average payment per day for each HHRG cannot be done because the payments are not itemized by HHRG and about half of episodes have more than 1 HHRG. We therefore computed the average payment per day, regardless of the HHRG.

For LUPA episodes, the payment depends on the number and the type of visits (physical therapy, occupational therapy, speech language pathology, skilled nursing, medical social services, or home health aide). These services

© 2026 Lin YL et al. *JAMA Network Open*

were identified using revenue center codes 042x, 043x, 044x, 055x, 056x, 057x, respectively. The standard payment was computed as the average Medicare payment per visit for each type of visit.

For RAP episodes, we did not include a payment because they are anticipated payments and do not represent the utilization at the time of payment. Such home health episodes comprised only 2.8% of total home health claims.

Steps for estimating standard payment:

1. Select claims from providers in the 11 control states between 2011 and 2018. Select claims with Medicare as the primary payer. Remove denied claims.
2. Adjust the payment amount to 2018 dollars using the Medical Care CPI. For the full and PEP home health episodes, use the claim-level payment amount since each visit is not paid separately. For LUPA episodes, use the revenue center payment amount which represents the payment per visit.
3. Compute the average payment per day (full or PEP episodes) or per visit (LUPA episodes).
4. Prorate the payment if the home health episode extended outside the chemotherapy episode.

Hospice

As per the *CMS Standardization Methodology For Allowed Amount – v.13 (updated August 2024),*[5] Medicare covers 5 hospice services:

1) Continuous Home Care (CHC)
2) Routine Home Care (RHC)
3) Inpatient Respite Care (IRC)
4) General Inpatient Care (GIC)
5) Physician/NP service

These services were identified using revenue center codes 0652, 0651, 0655, 0656, and 0657, respectively. For each service, the standard payment was computed as the average Medicare payment per unit of service.

Steps for estimating standard payment:

1. Select claims from providers in the 11 control states between 2011 and 2018. Select claims with Medicare as the primary payer. Remove denied claims.
2. Adjust the payment amount to 2018 dollars using the Medical Care CPI.
3. Compute the average payment per service unit for each type of service.
4. Prorate the payment if the hospice stay extended outside the chemotherapy episode.

Part D Drugs

We used the gross drug cost instead of the amount paid by Part D plan to calculate the standard payment for each drug because we believe the gross drug cost better represents the utilization. Part D event (PDE) records with zero Part D plan payment amount are those events occurring within the deductible phase. However, the deductible varies by plan. Also, because the penetration of the zero-deductible plans may be different in Maryland and control states, using the amount paid by Part D plan to approximate utilization could be biased. That is, beneficiaries in a state tending to have plans with deductibles would appear to utilize fewer medications compared to other states because the amounts paid by their Part D plans is zero before the deductible is reached.

We chose to compute the average gross cost for each drug's generic name instead of its National Drug Code (NDC). The drug price is market-based so there is variation in prices for drugs with the same active ingredient. If we had instead used the average gross cost for each NDC, the brand-name and generic drugs would have had different standard payment amounts, and thus, patients using brand-name drugs would have seemed to have a higher utilization than those who used generic drugs.

© 2026 Lin YL et al. *JAMA Network Open*

Steps for estimating standard payment:

1. Select PDE records between 2011 and 2018.
2. Adjust the gross cost amount to 2018 dollars using the Medical Care CPI.
3. Compute the average gross cost for each generic name, per unit dispensed.

© 2026 Lin YL et al. *JAMA Network Open*

eAppendix 3. Matching Strategy

Using the initial cohort (eTable 1), we performed a two-step match to identify comparable chemotherapy episodes in Maryland and control states, described below.

Step 1. Match Hospital Service Areas (HSAs) in Maryland and control states

We matched the HSAs on three HSA characteristics: urbanization in 2013 (pre-GBR), time trends in hospital Medicare payments between 2011 and 2013, and time trends in professional Medicare payments between 2011 and 2013.

The urbanization in each HSA was based on USDA Rural-Urban Commuting Area (RUCA) Codes. RUCA codes are at the zip code level. For each HSA, we calculated the proportion of zip codes with each RUCA code. For example, the Annapolis HSA in Maryland has 23 zip codes, 16 (69.6%) of the zip codes had a RUCA code of 1 (Metropolitan area core: primary flow within an urbanized area) in 2013 and 7 (30.4%) had a RUCA code of 2 (Metropolitan area high commuting: primary flow 30% or more to an urban area).

The time trends in hospital Medicare payments and professional Medicare payments between 2011 and 2013 were estimated by ordinary least square (OLS) regressions with log payments as the dependent variable and chemotherapy year as the independent variable, stratified by HSA without any adjustment.

Through an iterative process, we identified the best grouping of these three HSA characteristics for the coarsened exact match to ensure parallel trends of the payment outcome measures in the pre-GBR period (2011-2013). For urbanization in 2013, we categorized HSAs into three groups:

(1) High urbanization: > 95% of zip codes had RUCA code 1
(2) Moderate urbanization: ≤ 95% of zip codes had RUCA code 1 and > 80% of zip codes had RUCA code 1 or 2.
(3) Low urbanization: ≤ 80% of zip codes had RUCA code 1 or 2.

For the time trend in hospital payments between 2011 and 2013 (i.e., the coefficient of the chemotherapy year in the OLS regression), we categorized HSAs into two groups:

(1) -0.75 to -0.10
(2) -0.10 to 0.35

For the time trend in professional payments between 2011 and 2013 (i.e., the coefficient of the chemotherapy year in the OLS regression), we categorized HSAs into two groups:

(1) -0.2 to 0.0
(2) 0.0 to 0.4

In the initial cohort, there were 12 HSAs in Maryland and 279 HSAs in control states. Using the coarsened exact match with the groupings above, we matched the 12 Maryland HSAs to 140 control HSAs.

Step 2: Match chemotherapy episodes by treatment year, patient demographics, and clinical characteristics.

From the matched HSAs in Step 1, we identified three matched samples using propensity matching applicable to studying three different sets of outcome measures. Specifically, we utilized the 1:1 Greedy Nearest Neighbor Matching approach.

(1) **Main sample** for studying total episode payments, hospital payments, professional payments, all-cause hospitalization, all-cause emergency department (ED) visit, chemotherapy-related hospitalization, and chemotherapy-related ED visits: The propensity model included treatment year, patient age, sex, race and

© 2026 Lin YL et al. *JAMA Network Open*

ethnicity, dual eligibility, institutional status, disability index, Part D enrollment during the episode, Part B or D chemotherapy, cancer type, metastasis status, any prior chemotherapy episode, comorbidity using hierarchical condition category (HCC) groups, and zip code-level variables: social deprivation index and percent uninsured. Among these variables, treatment year and chemotherapy type (whether the episode initiating chemotherapy was a Part B or D drug) were exactly matched. In the initial cohort, there were 38,541 episodes from the 12 Maryland HSAs. Among them, 38,531 (99.97%) episodes were matched to 38,531 episodes in control HSAs. The patient characteristics and HSA-level characteristics before and after match are summarized in Table 1 and eTable 4.

(2) **Sub-sample 1** for studying timely receipt of presumed adjuvant chemotherapy within 60 days of curative-intent surgery: We followed methods used in the Oncology Care Model[6] evaluation and focused on episodes for patients who underwent curative surgery for colorectal, lung, or breast cancer in the 6 months preceding the episode initiation. These surgeries were identified from inpatient, outpatient, and carrier claims using ICD CPT codes listed in eTable 2. In the initial cohort, there were 2,259 such episodes in Maryland and 35,029 episodes in control states. In the 140 matched control HSAs, there were 22,541 episodes with curative surgery. After the propensity match, 2,104 (93.1%) episodes in Maryland HSAs were matched. The variables included in the propensity model are the same as those described in the Main sample above. However, in addition to treatment year and chemotherapy type (Part B or D), cancer type was also exactly matched. The patient characteristics and HSA-level characteristics before and after match are summarized in eTable 5.

(3) **Sub-sample 2** for studying end-of-life measures: no or late (within 3 days of death) hospice enrollment; more than one ED visit in the last 30 days of life; any intensive care unit (ICU) admission in the last 30 days of life; chemotherapy in the last 14 days of life: From the initial cohort, we identified 8,322 patients in Maryland HSAs and 139,649 patients in control states who died during the chemotherapy episode or within 90 days after the end of the episode. Then we selected those with continuous Parts A and B enrollment till death (8,312 and 139,470 patients in Maryland and control states, respectively). In the 140 matched HSAs, there were 91,169 patients. After the propensity match, 8,281 (99.6%) patients in Maryland were matched. The same matching approach described in the Main sample above was used with one modification: instead of "Part D enrollment during the episode", we included "Part D enrollment during the episode and through the date of death" in the propensity model. The patient characteristics and HSA-level characteristics before and after match are summarized in eTable 6.

© 2026 Lin YL et al. *JAMA Network Open*

eTable 2. ICD and CPT codes used for identifying curative surgeries for breast, lung, and colorectal cancers[1]

Type	Code	Definition
**Breast Cancer Surgery**		
ICD9	8521	Local excision of lesion of breast
	8522	Resection of quadrant of breast
	8523	Subtotal mastectomy
	8541	Unilateral simple mastectomy
	8542	Bilateral simple mastectomy
	8543	Unilateral extended simple mastectomy
	8544	Bilateral extended simple mastectomy
	8545	Unilateral radical mastectomy
	8546	Bilateral radical mastectomy
	8547	Unilateral extended radical mastectomy
	8548	Bilateral extended radical mastectomy
ICD10	0HTT0ZZ	Resection of Right Breast, Open Approach
	0HTU0ZZ	Resection of Left Breast, Open Approach
	0HTV0ZZ	Resection of Bilateral Breast, Open Approach
	0HBT0ZZ	Excision of Right Breast, Open Approach
	0HBT3ZZ	Excision of Right Breast, Percutaneous Approach
	0HBT7ZZ	Excision of Right Breast, Via Natural or Artificial Opening
	0HBT8ZZ	Excision of Right Breast, Via Natural or Artificial Opening Endoscopic
	0HBTXZZ	Excision of Right Breast, External Approach
	0HBU0ZZ	Excision of Left Breast, Open Approach
	0HBU3ZZ	Excision of Left Breast, Percutaneous Approach
	0HBU7ZZ	Excision of Left Breast, Via Natural or Artificial Opening
	0HBU8ZZ	Excision of Left Breast, Via Natural or Artificial Opening Endoscopic
	0HBUXZZ	Excision of Left Breast, External Approach
	0HBV0ZZ	Excision of Bilateral Breast, Open Approach
	0HBV3ZZ	Excision of Bilateral Breast, Percutaneous Approach
	0HBV7ZZ	Excision of Bilateral Breast, Via Natural or Artificial Opening
	0HBV8ZZ	Excision of Bilateral Breast, Via Natural or Artificial Opening Endoscopic
	0HBVXZZ	Excision of Bilateral Breast, External Approach
CPT	19120	Excision of cyst, fibroadenoma, or other benign or malignant tumor, aberrant breast tissue, duct lesion, nipple or areolar lesion
	19125	Excision of breast lesion identified by preoperative placement of radiological marker, open; single lesion
	19126	Excision of breast lesion identified by preoperative placement of radiological marker, open; each additional lesion separately identified by a preoperative radiological marker
	19160	Mastectomy, partial (eg, lumpectomy, tylectomy, quadrantectomy, segmentectomy)
	19162	Mastectomy, partial (eg, lumpectomy, tylectomy, quadrantectomy, segmentectomy); with axillary lymphadenectomy
	19180	Mastectomy, simple, complete

© 2026 Lin YL et al. *JAMA Network Open*

	19182	Mastectomy, subcutaneous
	19200	Mastectomy, radical, including pectoral muscles, axillary lymph nodes
	19220	Mastectomy, radical, including pectoral muscles, axillary and internal mammary lymph nodes (Urban type operation)
	19240	Mastectomy, modified radical, including axillary lymph nodes, with or without pectoralis minor muscle, but excluding pectoralis major muscle
	19301	Mastectomy, partial (eg, lumpectomy, tylectomy, quadrantectomy, segmentectomy)
	19302	Mastectomy, partial (eg, lumpectomy, tylectomy, quadrantectomy, segmentectomy); with axillary lymphadenectomy
	19303	Mastectomy, simple, complete
	19304	Mastectomy, subcutaneous
	19305	Mastectomy, radical, including pectoral muscles, axillary lymph nodes
	19306	Mastectomy, radical, including pectoral muscles, axillary and internal mammary lymph nodes (Urban type operation)
	19307	Mastectomy, modified radical, including axillary lymph nodes, with or without pectoralis minor muscle, but excluding pectoralis major muscle
**Colorectal Cancer Surgery**		
ICD9	1731	Laparoscopic multiple segmental resection of large intestine
	1732	Laparoscopic cecectomy
	1733	Laparoscopic right hemicolectomy
	1734	Laparoscopic resection of transverse colon
	1735	Laparoscopic left hemicolectomy
	1736	Laparoscopic sigmoidectomy
	1739	Other laparoscopic partial excision of large intestine
	4571	Open and other multiple segmental resection of large intestine
	4572	Open and other cecectomy
	4573	Open and other right hemicolectomy
	4574	Open and other resection of transverse colon
	4575	Open and other left hemicolectomy
	4576	Open and other sigmoidectomy
	4579	Other and unspecified partial excision of large intestine
	4581	Laparoscopic total intra-abdominal colectomy
	4582	Open total intra-abdominal colectomy
	4583	Other and unspecified total intra-abdominal colectomy
	4604	Resection of exteriorized segment of large intestine
	4840	Pull-through resection of rectum, not otherwise specified
	4841	Soave submucosal resection of rectum
	4842	Laparoscopic pull-through resection of rectum
	4843	Open pull-through resection of rectum
	4849	Other pull-through resection of rectum
	4850	Abdominoperineal resection of the rectum, not otherwise specified
	4851	Laparoscopic abdominoperineal resection of the rectum
	4852	Open abdominoperineal resection of the rectum
	4859	Other abdominoperineal resection of the rectum
	4861	Transsacral rectosigmoidectomy

© 2026 Lin YL et al. *JAMA Network Open*

	4862	Anterior resection of rectum with synchronous colostomy
	4863	Other anterior resection of rectum
	4864	Posterior resection of rectum
	4865	Duhamel resection of rectum
	4869	Other resection of rectum
ICD10	0DBE0ZZ	Excision of Large Intestine, Open Approach
	0DBE3ZZ	Excision of Large Intestine, Percutaneous Approach
	0DBE4ZZ	Excision of Large Intestine, Percutaneous Endoscopic Approach
	0DBE7ZZ	Excision of Large Intestine, Via Natural or Artificial Opening
	0DBE8ZZ	Excision of Large Intestine, Via Natural or Artificial Opening Endoscopic
	0DBF0ZZ	Excision of Right Large Intestine, Open Approach
	0DBF3ZZ	Excision of Right Large Intestine, Percutaneous Approach
	0DBF4ZZ	Excision of Right Large Intestine, Percutaneous Endoscopic Approach
	0DBF7ZZ	Excision of Right Large Intestine, Via Natural or Artificial Opening
	0DBF8ZZ	Excision of Right Large Intestine, Via Natural or Artificial Opening Endoscopic
	0DBG0ZZ	Excision of Left Large Intestine, Open Approach
	0DBG3ZZ	Excision of Left Large Intestine, Percutaneous Approach
	0DBG4ZZ	Excision of Left Large Intestine, Percutaneous Endoscopic Approach
	0DBG7ZZ	Excision of Left Large Intestine, Via Natural or Artificial Opening
	0DBG8ZZ	Excision of Left Large Intestine, Via Natural or Artificial Opening Endoscopic
	0DBGFZZ	Excision of Left Large Intestine, Via Natural or Artificial Opening With Percutaneous Endoscopic Assistance
	0DBH0ZZ	Excision of Cecum, Open Approach
	0DBH3ZZ	Excision of Cecum, Percutaneous Approach
	0DBH4ZZ	Excision of Cecum, Percutaneous Endoscopic Approach
	0DBH7ZZ	Excision of Cecum, Via Natural or Artificial Opening
	0DBH8ZZ	Excision of Cecum, Via Natural or Artificial Opening Endoscopic
	0DBK0ZZ	Excision of Ascending Colon, Open Approach
	0DBK3ZZ	Excision of Ascending Colon, Percutaneous Approach
	0DBK4ZZ	Excision of Ascending Colon, Percutaneous Endoscopic Approach
	0DBK7ZZ	Excision of Ascending Colon, Via Natural or Artificial Opening
	0DBK8ZZ	Excision of Ascending Colon, Via Natural or Artificial Opening Endoscopic
	0DBL0ZZ	Excision of Transverse Colon, Open Approach
	0DBL3ZZ	Excision of Transverse Colon, Percutaneous Approach
	0DBL4ZZ	Excision of Transverse Colon, Percutaneous Endoscopic Approach
	0DBL7ZZ	Excision of Transverse Colon, Via Natural or Artificial Opening
	0DBL8ZZ	Excision of Transverse Colon, Via Natural or Artificial Opening Endoscopic
	0DBLFZZ	Excision of Transverse Colon, Via Natural or Artificial Opening With Percutaneous Endoscopic Assistance
	0DBM0ZZ	Excision of Descending Colon, Open Approach
	0DBM3ZZ	Excision of Descending Colon, Percutaneous Approach
	0DBM4ZZ	Excision of Descending Colon, Percutaneous Endoscopic Approach
	0DBM7ZZ	Excision of Descending Colon, Via Natural or Artificial Opening
	0DBM8ZZ	Excision of Descending Colon, Via Natural or Artificial Opening Endoscopic

© 2026 Lin YL et al. *JAMA Network Open*

	0DBMFZZ	Excision of Descending Colon, Via Natural or Artificial Opening With Percutaneous Endoscopic Assistance
	0DBN0ZZ	Excision of Sigmoid Colon, Open Approach
	0DBN3ZZ	Excision of Sigmoid Colon, Percutaneous Approach
	0DBN4ZZ	Excision of Sigmoid Colon, Percutaneous Endoscopic Approach
	0DBN7ZZ	Excision of Sigmoid Colon, Via Natural or Artificial Opening
	0DBN8ZZ	Excision of Sigmoid Colon, Via Natural or Artificial Opening Endoscopic
	0DBNFZZ	Excision of Sigmoid Colon, Via Natural or Artificial Opening With Percutaneous Endoscopic Assistance
	0DBP0ZZ	Excision of Rectum, Open Approach
	0DBP3ZZ	Excision of Rectum, Percutaneous Approach
	0DBP4ZZ	Excision of Rectum, Percutaneous Endoscopic Approach
	0DBP7ZZ	Excision of Rectum, Via Natural or Artificial Opening
	0DBP8ZZ	Excision of Rectum, Via Natural or Artificial Opening Endoscopic
	0DTE0ZZ	Resection of Large Intestine, Open Approach
	0DTE4ZZ	Resection of Large Intestine, Percutaneous Endoscopic Approach
	0DTE7ZZ	Resection of Large Intestine, Via Natural or Artificial Opening
	0DTE8ZZ	Resection of Large Intestine, Via Natural or Artificial Opening Endoscopic
	0DTF0ZZ	Resection of Right Large Intestine, Open Approach
	0DTF4ZZ	Resection of Right Large Intestine, Percutaneous Endoscopic Approach
	0DTF7ZZ	Resection of Right Large Intestine, Via Natural or Artificial Opening
	0DTF8ZZ	Resection of Right Large Intestine, Via Natural or Artificial Opening Endoscopic
	0DTG0ZZ	Resection of Left Large Intestine, Open Approach
	0DTG4ZZ	Resection of Left Large Intestine, Percutaneous Endoscopic Approach
	0DTG7ZZ	Resection of Left Large Intestine, Via Natural or Artificial Opening
	0DTG8ZZ	Resection of Left Large Intestine, Via Natural or Artificial Opening Endoscopic
	0DTGFZZ	Resection of Left Large Intestine, Via Natural or Artificial Opening With Percutaneous Endoscopic Assistance
	0DTH0ZZ	Resection of Cecum, Open Approach
	0DTH4ZZ	Resection of Cecum, Percutaneous Endoscopic Approach
	0DTH7ZZ	Resection of Cecum, Via Natural or Artificial Opening
	0DTH8ZZ	Resection of Cecum, Via Natural or Artificial Opening Endoscopic
	0DTK0ZZ	Resection of Ascending Colon, Open Approach
	0DTK4ZZ	Resection of Ascending Colon, Percutaneous Endoscopic Approach
	0DTK7ZZ	Resection of Ascending Colon, Via Natural or Artificial Opening
	0DTK8ZZ	Resection of Ascending Colon, Via Natural or Artificial Opening Endoscopic
	0DTL0ZZ	Resection of Transverse Colon, Open Approach
	0DTL4ZZ	Resection of Transverse Colon, Percutaneous Endoscopic Approach
	0DTL7ZZ	Resection of Transverse Colon, Via Natural or Artificial Opening
	0DTL8ZZ	Resection of Transverse Colon, Via Natural or Artificial Opening Endoscopic
	0DTLFZZ	Resection of Transverse Colon, Via Natural or Artificial Opening With Percutaneous Endoscopic Assistance
	0DTMFZZ	Resection of Descending Colon, Via Natural or Artificial Opening With Percutaneous Endoscopic Assistance
	0DTM0ZZ	Resection of Descending Colon, Open Approach

© 2026 Lin YL et al. *JAMA Network Open*

	0DTM4ZZ	Resection of Descending Colon, Percutaneous Endoscopic Approach
	0DTM7ZZ	Resection of Descending Colon, Via Natural or Artificial Opening
	0DTM8ZZ	Resection of Descending Colon, Via Natural or Artificial Opening Endoscopic
	0DTN0ZZ	Resection of Sigmoid Colon, Open Approach
	0DTN4ZZ	Resection of Sigmoid Colon, Percutaneous Endoscopic Approach
	0DTN7ZZ	Resection of Sigmoid Colon, Via Natural or Artificial Opening
	0DTN8ZZ	Resection of Sigmoid Colon, Via Natural or Artificial Opening Endoscopic
	0DTNFZZ	Resection of Sigmoid Colon, Via Natural or Artificial Opening With Percutaneous Endoscopic Assistance
	0DTP0ZZ	Resection of Rectum, Open Approach
	0DTP4ZZ	Resection of Rectum, Percutaneous Endoscopic Approach
	0DTP7ZZ	Resection of Rectum, Via Natural or Artificial Opening
	0DTP8ZZ	Resection of Rectum, Via Natural or Artificial Opening Endoscopic
CPT	44140	Colectomy, partial; with anastomosis
	44141	Colectomy, partial; with skin level cecostomy or colostomy
	44143	Colectomy, partial; with end colostomy and closure of distal segment (Hartmann type procedure)
	44144	Colectomy, partial; with resection, with colostomy or ileostomy and creation of mucofistula
	44145	Colectomy, partial; with coloproctostomy (low pelvic anastomosis)
	44146	Colectomy, partial; with coloproctostomy (low pelvic anastomosis), with colostomy
	44147	Colectomy, partial; abdominal and transanal approach
	44150	Colectomy, total, abdominal, without proctectomy; with ileostomy or ileoproctostomy
	44151	Colectomy, total, abdominal, without proctectomy; with continent ileostomy
	44155	Colectomy, total, abdominal, with proctectomy; with ileostomy
	44156	Colectomy, total, abdominal, with proctectomy; with continent ileostomy
	44157	Colectomy, total, abdominal, with proctectomy; with ileoanal anastomosis, includes loop ileostomy, and rectal mucosectomy, when performed
	44158	Colectomy, total, abdominal, with proctectomy; with ileoanal anastomosis, creation of ileal reservoir (S or J), includes loop ileostomy, and rectal mucosectomy, when performed
	44160	Colectomy, partial, with removal of terminal ileum with ileocolostomy
	44204	Laparoscopy, surgical; colectomy, partial, with anastomosis
	44205	Laparoscopy, surgical; colectomy, partial, with removal of terminal ileum with ileocolostomy
	44206	Laparoscopy, surgical; colectomy, partial, with end colostomy and closure of distal segment (Hartmann type procedure)
	44207	Laparoscopy, surgical; colectomy, partial, with anastomosis, with coloproctostomy (low pelvic anastomosis)
	44208	Laparoscopy, surgical; colectomy, partial, with anastomosis, with coloproctostomy (low pelvic anastomosis) with colostomy
	44210	Laparoscopy, surgical; colectomy, total, abdominal, without proctectomy, with ileostomy or ileoproctostomy

© 2026 Lin YL et al. *JAMA Network Open*

	44211	Laparoscopy, surgical; colectomy, total, abdominal, with proctectomy, with ileoanal anastomosis, creation of ileal reservoir (S or J), with loop ileostomy, includes rectal mucosectomy, when performed
	44212	Laparoscopy, surgical; colectomy, total, abdominal, with proctectomy, with ileostomy
	44213	Laparoscopy, surgical, mobilization (take-down) of splenic flexure performed in conjunction with partial colectomy
	45110	Proctectomy; complete, combined abdominoperineal, with colostomy
	45111	Proctectomy; partial resection of rectum, transabdominal approach
	45112	Proctectomy, combined abdominoperineal, pull-through procedure (eg, colo-anal anastomosis)
	45113	Proctectomy, partial, with rectal mucosectomy, ileoanal anastomosis, creation of ileal reservoir (S or J), with or without loop ileostomy
	45114	Proctectomy, partial, with anastomosis; abdominal and transsacral approach
	45116	Proctectomy, partial, with anastomosis; transsacral approach only (Kraske type)
	45119	Proctectomy, combined abdominoperineal pull-through procedure (eg, colo-anal anastomosis), with creation of colonic reservoir (eg, J-pouch), with diverting enterostomy when performed
	45120	Proctectomy, complete (for congenital megacolon), abdominal and perineal approach; with pull-through procedure and anastomosis (eg, Swenson, Duhamel, or Soave type operation)
	45121	Proctectomy, complete (for congenital megacolon), abdominal and perineal approach; with subtotal or total colectomy, with multiple biopsies
	45123	Proctectomy, partial, without anastomosis, perineal approach
	45126	Pelvic exenteration for colorectal malignancy, with proctectomy (with or without colostomy), with removal of bladder and ureteral transplantations, and/or hysterectomy, or cervicectomy, with or without removal of tube(s), with or without removal of ovary(s), or any combination thereof
**Lung Cancer Surgery**		
**ICD9**	3230	Thoracoscopic segmental resection of lung
	3239	Other and unspecified segmental resection of lung
	3241	Thoracoscopic lobectomy of lung
	3249	Other lobectomy of lung
	3250	Thoracoscopic pneumonectomy
	3259	Other and unspecified pneumonectomy
	326	Radical dissection of thoracic structures
**ICD10**	0BTC0ZZ	Resection of Right Upper Lung Lobe, Open Approach
	0BTC4ZZ	Resection of Right Upper Lung Lobe, Percutaneous Endoscopic Approach
	0BTD0ZZ	Resection of Right Middle Lung Lobe, Open Approach
	0BTD4ZZ	Resection of Right Middle Lung Lobe, Percutaneous Endoscopic Approach
	0BTF0ZZ	Resection of Right Lower Lung Lobe, Open Approach
	0BTF4ZZ	Resection of Right Lower Lung Lobe, Percutaneous Endoscopic Approach
	0BTG0ZZ	Resection of Left Upper Lung Lobe, Open Approach
	0BTG4ZZ	Resection of Left Upper Lung Lobe, Percutaneous Endoscopic Approach
	0BTH0ZZ	Resection of Lung Lingula, Open Approach

© 2026 Lin YL et al. *JAMA Network Open*

0BTH4ZZ	Resection of Lung Lingula, Percutaneous Endoscopic Approach
0BTJ0ZZ	Resection of Left Lower Lung Lobe, Open Approach
0BTJ4ZZ	Resection of Left Lower Lung Lobe, Percutaneous Endoscopic Approach
0BTK0ZZ	Resection of Right Lung, Open Approach
0BTK4ZZ	Resection of Right Lung, Percutaneous Endoscopic Approach
0BTL0ZZ	Resection of Left Lung, Open Approach
0BTL4ZZ	Resection of Left Lung, Percutaneous Endoscopic Approach
0BTM0ZZ	Resection of Bilateral Lungs, Open Approach
0BTM4ZZ	Resection of Bilateral Lungs, Percutaneous Endoscopic Approach
0BBC0ZZ	Excision of Right Upper Lung Lobe, Open Approach
0BBC3ZZ	Excision of Right Upper Lung Lobe, Percutaneous Approach
0BBC4ZZ	Excision of Right Upper Lung Lobe, Percutaneous Endoscopic Approach
0BBC7ZZ	Excision of Right Upper Lung Lobe, Via Natural or Artificial Opening
0BBC8ZZ	Excision of Right Upper Lung Lobe, Via Natural or Artificial Opening Endoscopic
0BBD0ZZ	Excision of Right Middle Lung Lobe, Open Approach
0BBD3ZZ	Excision of Right Middle Lung Lobe, Percutaneous Approach
0BBD4ZZ	Excision of Right Middle Lung Lobe, Percutaneous Endoscopic Approach
0BBD7ZZ	Excision of Right Middle Lung Lobe, Via Natural or Artificial Opening
0BBD8ZZ	Excision of Right Middle Lung Lobe, Via Natural or Artificial Opening Endoscopic
0BBF0ZZ	Excision of Right Lower Lung Lobe, Open Approach
0BBF3ZZ	Excision of Right Lower Lung Lobe, Percutaneous Approach
0BBF4ZZ	Excision of Right Lower Lung Lobe, Percutaneous Endoscopic Approach
0BBF7ZZ	Excision of Right Lower Lung Lobe, Via Natural or Artificial Opening
0BBF8ZZ	Excision of Right Lower Lung Lobe, Via Natural or Artificial Opening Endoscopic
0BBG0ZZ	Excision of Left Upper Lung Lobe, Open Approach
0BBG3ZZ	Excision of Left Upper Lung Lobe, Percutaneous Approach
0BBG4ZZ	Excision of Left Upper Lung Lobe, Percutaneous Endoscopic Approach
0BBG7ZZ	Excision of Left Upper Lung Lobe, Via Natural or Artificial Opening
0BBG8ZZ	Excision of Left Upper Lung Lobe, Via Natural or Artificial Opening Endoscopic
0BBH0ZZ	Excision of Lung Lingula, Open Approach
0BBH3ZZ	Excision of Lung Lingula, Percutaneous Approach
0BBH4ZZ	Excision of Lung Lingula, Percutaneous Endoscopic Approach
0BBH7ZZ	Excision of Lung Lingula, Via Natural or Artificial Opening
0BBH8ZZ	Excision of Lung Lingula, Via Natural or Artificial Opening Endoscopic
0BBJ0ZZ	Excision of Left Lower Lung Lobe, Open Approach
0BBJ3ZZ	Excision of Left Lower Lung Lobe, Percutaneous Approach
0BBJ4ZZ	Excision of Left Lower Lung Lobe, Percutaneous Endoscopic Approach
0BBJ7ZZ	Excision of Left Lower Lung Lobe, Via Natural or Artificial Opening
0BBJ8ZZ	Excision of Left Lower Lung Lobe, Via Natural or Artificial Opening Endoscopic
0BBK0ZZ	Excision of Right Lung, Open Approach
0BBK3ZZ	Excision of Right Lung, Percutaneous Approach
0BBK4ZZ	Excision of Right Lung, Percutaneous Endoscopic Approach

© 2026 Lin YL et al. *JAMA Network Open*

	0BBK7ZZ	Excision of Right Lung, Via Natural or Artificial Opening
	0BBK8ZZ	Excision of Right Lung, Via Natural or Artificial Opening Endoscopic
	0BBL0ZZ	Excision of Left Lung, Open Approach
	0BBL3ZZ	Excision of Left Lung, Percutaneous Approach
	0BBL4ZZ	Excision of Left Lung, Percutaneous Endoscopic Approach
	0BBL7ZZ	Excision of Left Lung, Via Natural or Artificial Opening
	0BBL8ZZ	Excision of Left Lung, Via Natural or Artificial Opening Endoscopic
	0BBM0ZZ	Excision of Bilateral Lungs, Open Approach
	0BBM3ZZ	Excision of Bilateral Lungs, Percutaneous Approach
	0BBM4ZZ	Excision of Bilateral Lungs, Percutaneous Endoscopic Approach
	0BBM7ZZ	Excision of Bilateral Lungs, Via Natural or Artificial Opening
	0BBM8ZZ	Excision of Bilateral Lungs, Via Natural or Artificial Opening Endoscopic
CPT	32440	Removal of lung, pneumonectomy;
	32442	Removal of lung, pneumonectomy; with resection of segment of trachea followed by broncho-tracheal anastomosis (sleeve pneumonectomy)
	32445	Removal of lung, pneumonectomy; extrapleural
	32480	Removal of lung, other than pneumonectomy; single lobe (lobectomy)
	32482	Removal of lung, other than pneumonectomy; 2 lobes (bilobectomy)
	32484	Removal of lung, other than pneumonectomy; single segment (segmentectomy)
	32486	Removal of lung, other than pneumonectomy; with circumferential resection of segment of bronchus followed by broncho-bronchial anastomosis (sleeve lobectomy)
	32663	Thoracoscopy, surgical; with lobectomy (single lobe)
	32500	Removal of lung, other than total pneumonectomy; wedge resection, single or multiple
	32505	Thoracotomy; with therapeutic wedge resection (eg, mass, nodule), initial
	32506	Thoracotomy; with therapeutic wedge resection (eg, mass or nodule), each additional resection, ipsilateral (List separately in addition to code for primary procedure)
	32507	Thoracotomy; with diagnostic wedge resection followed by anatomic lung resection (List separately in addition to code for primary procedure)
	32657	Thoracoscopy, surgical; with wedge resection of lung, single, or multiple
	32666	Thoracoscopy, surgical; with therapeutic wedge resection (eg, mass, nodule), initial unilateral
	32667	Thoracoscopy, surgical; with therapeutic wedge resection (eg, mass or nodule), each additional resection, ipsilateral (List separately in addition to code for primary procedure)
	32668	Thoracoscopy, surgical; with diagnostic wedge resection followed by anatomic lung resection (List separately in addition to code for primary procedure)
	32488	Removal of lung, other than pneumonectomy; with all remaining lung following previous removal of a portion of lung (completion pneumonectomy)
	32669	Thorascopy, surgical; with removal of a single lung segment (segmentectomy)
	32670	Thoracoscopy, surgical; with removal of two lobes (bilobectomy)
	32671	Thoracoscopy, surgical; with removal of lung (pneumonectomy)

© 2026 Lin YL et al. *JAMA Network Open*

eAppendix 4. Model for Estimating Adjusted Means

Trends of adjusted means for each outcome measure shown in Figures 1-3 were estimated from the following model specification:

$$O_{it} = \alpha_0 + \alpha_n I_{it} + \alpha_2 X_i + \varepsilon_{it}$$

where O_{it} is the outcome measure for chemotherapy episode i in year t. For the payment outcome measures, we winsorized the data at the 99th percentile and used the gamma distribution with a log link function. For binary outcome measures, we used the linear probability model by fitting the normal distribution. I_{it} is a set of 15 indicators representing the 16 data points in the figures: 8 for Maryland and 8 for control states (one for each year in each group). X_i is a set of covariates for episode i; ε_{it} is the error term.

Covariates included patient age, sex, race and ethnicity, dual eligibility, institutional status, disability index, continuous Part D enrollment, whether initiating chemotherapy is Part B or D chemotherapy, cancer type, metastasis status, any prior chemotherapy episode in the past 12 months, and comorbidity using HCC groups. We also included zip code-level social deprivation index and percent uninsured, as well as year-specific HSA level variables: number of billing oncologists per 10,000 elderly population, oncology practice-defined Herfindahl–Hirschman index (HHI),[7] number of hospital-owned oncology practices, total number of beds, percent of the HSA's hospitals that are 340B hospitals, and percent of the HSA's hospitals that are teaching hospitals.

eAppendix 5. Estimating Difference-in-Differences (DiD) and Testing the Parallel Trends Assumption

E.1 Primary DiD Model

The DiD results presented in the right-most column of Table 2 were estimated from the following model specification:

$$O_{it} = \alpha_0 + \alpha_1 GBR_i + \alpha_2 X_i + \alpha_3 L_l + \alpha_4 Y_t + \varepsilon_{it}$$

where GBR_i is a 0/1 indicator for a chemotherapy episode in Maryland after 2014's GBR implementation; X_i is a set of covariates for episode i; L_l is a set of HSA 0/1 fixed effect indicators; Y_t is a set of 0/1 indicators for each year t between 2011 and 2018; and ε_{it} is the error term. The coefficient α_1 represents the DiD estimate, as the differential post-period changes in Maryland relative to control states. The covariates X_i are the same as those described in Appendix D.

E.2 Sensitivity DiD Analysis Excluding GBR's Implementation Year

Sensitivity analyses excluding GBR's implementation year allows for a 12-month "washout" period under the assumption that practice changes in response to GBR may take time. These results were estimated from the following model specification:

$$O_{it} = \alpha_0 + \alpha_1 GBR_i + \alpha_2 GBR_{i2014} + \alpha_3 X_i + \alpha_4 L_l + \alpha_5 Y_t + \varepsilon_{it}$$

where GBR_i is a 0/1 indicator for a chemotherapy episode in Maryland between 2015 and 2018; GBR_{i2014} is a 0/1 indicator for chemotherapy episode in Maryland at the year of 2014. This term isolates the potential impact from GBR during the first year of implementation. X_i is a set of covariates for episode i; L_l is a set of HSA 0/1 fixed effect indicators; Y_t is a set of 0/1 indicators for the year t between 2011-2018; ε_{it} is the error term. The coefficient α_1 represents the DiD estimate, as the differential post-period changes in Maryland relative to control states.

E.3 Testing the Parallel Trends Assumption

For the DiD model above to produce an unbiased estimate, the outcome variable should exhibit similar time trends for Maryland and the control states during the 2011-2013 pre-period. This assumption of parallel trends was tested by estimating the following model specification:

$$O_{it} = \alpha_0 + \alpha_T \Sigma GBR_{iT} + \alpha_2 X_i + \alpha_3 L_l + \alpha_4 Y_t + \alpha_5 MD_{it} * LY_t + \varepsilon_{it}$$

where ΣGBR_{iT} is a set of 5 indicators for a chemotherapy episode in Maryland at year T post GBR implementation (2014 to 2018); X_i is a set of covariates for episode i; L_l is a set of HSA 0/1 fixed effect indicators; Y_t is a set of 0/1 indicators for the year t between 2011-2018; MD_{it} is a 0/1 indicator for episode i in Maryland at year t; LY_t is the linear time trend ranging from 1 to 8, respectively representing 2011 to 2018. The $MD_{it} * LY_t$ term represents the difference in slopes between Maryland and control groups before the GBR implementation.[8] A non-significant coefficient α_5 means the there is no evidence of differential pre-trends. The results of this test are summarized in eTable 3.

E.4 Estimating Event Study DiDs for Each Year

An "event study" approach to the DiD model assesses whether the effect of GBR varied over time after implementation. The results presented in eFigure 1 were estimated from the following model specification:

$$O_{it} = \alpha_0 + \alpha_t \Sigma GBR_{it} + \alpha_2 X_i + \alpha_3 L_l + \alpha_4 Y_t + \varepsilon_{it}$$

where ΣGBR_{it} is a set of 7 indicators for a chemotherapy episode in Maryland at year t. The year before the GBR implementation, 2013, is the omitted category that served as the reference. X_i is a set of covariates for episode *i*; L_l is a set of HSA 0/1 fixed effect indicators; Y_t is a set of 0/1 indicators for the year *t* between 2011-2018*;* ε_{it} is the error term. The coefficients α_t represent the year-specific DiDs which are the differential changes in Maryland relative to control states in each particular year relative to 2013.[9] The coefficients for 2011 and 2012 represent differences in the trends prior to implementation, while the coefficients for 2014 through 2018 represent potential variability over time within the aggregate DiD estimate.

© 2026 Lin YL et al. *JAMA Network Open*

eTable 3. Test for parallel trends assumption

Outcome Variable	Estimate for differential trend	p value
Total episode payments	-0.0185	0.0927
Hospital payments	-0.0233	0.2170
Professional payments	-0.0334	0.1237
Chemotherapy payments	-0.0954	< 0.0001
Part B chemotherapy payments	-0.0898	0.0029
All-cause hospitalization	0.0045	0.4972
All-cause ED visit	-0.0058	0.3097
Chemotherapy-related hospitalization	0.0088	0.1299
Chemotherapy-related ED visit	0.0000	0.9964
Timely receipt of chemotherapy	-0.0008	0.9793
No or late* hospice enrollment	-0.0335	0.0510
> 1 ED visit in last 30 days of life	-0.0206	0.1074
ICU visit in last 30 days of life	-0.0283	0.0507
Chemotherapy in last 14 days of life	0.0004	0.9729

*"Late" means hospice enrollment within 3 days of death.
All models were adjusted for patient age, sex, race and ethnicity, dual eligibility, institutional status, disability index, Part D enrollment, Part B or D chemotherapy, cancer type, metastasis status, any prior chemotherapy episode, comorbidity using HCC groups, zip code-level social deprivation index and percent uninsured, and year-specific HSA level variables: number of billing oncologists per 10,000 elderly population, oncology practice-defined HHI, number of hospital-owned oncology practices, total number of beds, percent of 340B hospitals, and percent of teaching hospitals. The chemotherapy year and HSA fixed effects were also included.

© 2026 Lin YL et al. *JAMA Network Open*

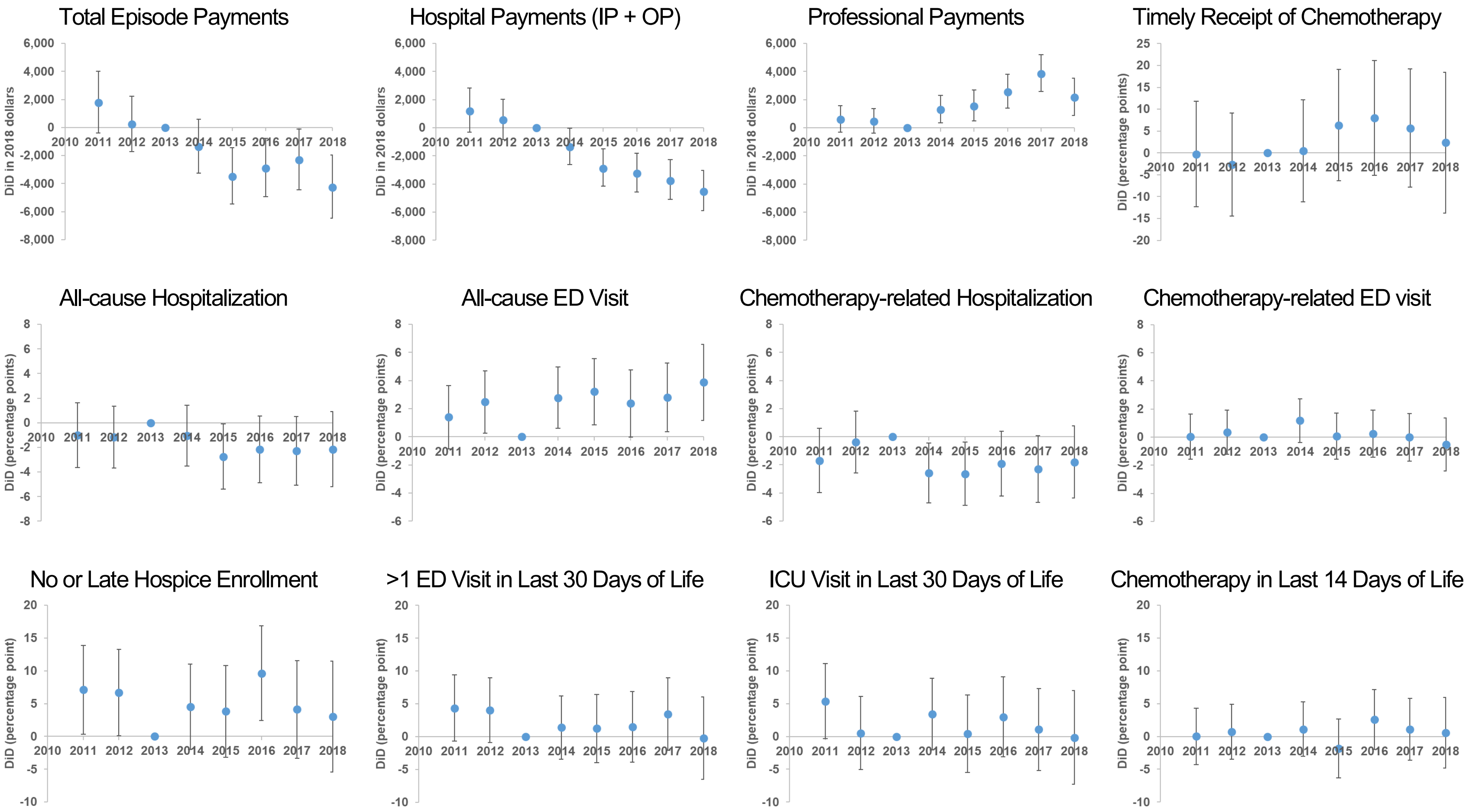

eFigure. Difference-in-differences each year with the year 2013 as the reference.

For the three payment outcomes, the Y-axis is the DiD estimate in 2018 dollars; for the other outcomes, the DiD estimate is in percentage points. All models were adjusted for patient age, sex, race and ethnicity, dual eligibility, institutional status, disability index, Part D enrollment, Part B or D chemotherapy, cancer type, metastasis status, any prior chemotherapy episode, comorbidity using HCC groups, zip code-level social deprivation index and percent uninsured, and year-specific HSA level variables: number of billing oncologists per 10,000 elderly population, oncology practice-defined HHI, number of hospital-owned oncology practices, total number of beds, percent 340B hospitals, and percent teaching hospitals. Year (based on chemotherapy initiation) and HSA fixed effects were also included. IP = inpatient; OP = outpatient.

© 2026 Lin YL et al. *JAMA Network Open*

eAppendix 6. Description of the Matched Samples

eTable 4. Comorbidity for chemotherapy episodes before and after matching in Maryland and control states – main sample

	Before match			After match		
Hierarchical Condition Category	Maryland n= 38,541	Control states n= 640,564	ASD	Maryland n= 38,531	Control states n= 38,531	ASD
	n (%)			n (%)		
Septicemia, Sepsis, Systemic Inflammatory Response Syndrome/Shock	3062 (7.9)	50092 (7.8)	0.00	3060 (7.9)	2488 (6.5)	0.06
HIV / AIDS / Opportunistic Infections	743 (1.9)	10682 (1.7)	0.02	743 (1.9)	591 (1.5)	0.03
Protein-Calorie Malnutrition	2019 (5.2)	46534 (7.3)	0.08	2019 (5.2)	2222 (5.8)	0.02
Morbid Obesity	1770 (4.6)	31676 (4.9)	0.02	1770 (4.6)	1838 (4.8)	0.01
Other Significant Endocrine and Metabolic Disorders	4476 (11.6)	83604 (13.1)	0.04	4474 (11.6)	4843 (12.6)	0.03
End-Stage Liver Disease	749 (1.9)	15009 (2.3)	0.03	749 (1.9)	794 (2.1)	0.01
Other GI diseases (Chronic Hepatitis, Chronic Pancreatitis, Inflammatory Bowel Disease)	1356 (3.5)	21630 (3.4)	0.01	1356 (3.5)	1138 (3.0)	0.03
Intestinal Obstruction / Perforation	2538 (6.6)	42191 (6.6)	0.00	2538 (6.6)	2249 (5.8)	0.03
Bone / Joint / Muscle Infections / Necrosis	768 (2.0)	13371 (2.1)	0.01	768 (2.0)	743 (1.9)	0.00
Rheumatoid Arthritis and Inflammatory Connective Tissue Disease	2480 (6.4)	50161 (7.8)	0.05	2480 (6.4)	2810 (7.3)	0.03
Hematological Disorders	9194 (23.9)	163242 (25.5)	0.04	9194 (23.9)	9182 (23.8)	0.00
Disorders of Immunity	7351 (19.1)	140090 (21.9)	0.07	7351 (19.1)	7611 (19.8)	0.02
Drug/Alcohol Dependence	1201 (3.1)	18863 (2.9)	0.01	1201 (3.1)	1044 (2.7)	0.02
Major psychiatric disorder	2689 (7.0)	48237 (7.5)	0.02	2689 (7.0)	2523 (6.5)	0.02
Spinal Cord Injury / Paralytic disorders	1107 (2.9)	18513 (2.9)	0.00	1107 (2.9)	1022 (2.7)	0.01
Neurological disorders affecting motor function	2064 (5.4)	38040 (5.9)	0.03	2063 (5.4)	2066 (5.4)	0.00
Other neurologic disorders (Multiple Sclerosis, Seizure, Coma, Brain Compression / Anoxic Damage)	1963 (5.1)	34267 (5.3)	0.01	1962 (5.1)	1947 (5.1)	0.00
Respirator Dependence / Tracheostomy Status	292 (0.8)	5511 (0.9)	0.01	292 (0.8)	286 (0.7)	0.00
Cardio-respiratory failure	2634 (6.8)	50176 (7.8)	0.04	2633 (6.8)	2655 (6.9)	0.00
Congestive Heart Failure	7484 (19.4)	134277 (21.0)	0.04	7483 (19.4)	7369 (19.1)	0.01
Acute Myocardial Infarction / Angina / Other Acute Ischemic Heart Disease	2674 (6.9)	55317 (8.6)	0.06	2674 (6.9)	2953 (7.7)	0.03
Specified Heart Arrhythmias	7400 (19.2)	134768 (21.0)	0.05	7399 (19.2)	7235 (18.8)	0.01

Cerebrovascular insult	2781 (7.2)	39167 (6.1)	0.04	2780 (7.2)	2193 (5.7)	0.06
Severe peripheral vascular disease	3036 (7.9)	51136 (8.0)	0.00	3035 (7.9)	2945 (7.6)	0.01
Vascular Disease	9473 (24.6)	168669 (26.3)	0.04	9469 (24.6)	9493 (24.6)	0.00
Cystic Fibrosis / Fibrosis of Lung and Other Chronic Lung Disorders	1516 (3.9)	24796 (3.9)	0.00	1516 (3.9)	1552 (4.0)	0.00
Chronic Obstructive Pulmonary Disease	9539 (24.8)	181629 (28.4)	0.08	9539 (24.8)	9509 (24.7)	0.00
Pneumonia	1577 (4.1)	29154 (4.6)	0.02	1576 (4.1)	1512 (3.9)	0.01
Exudative Macular Degeneration	746 (1.9)	12056 (1.9)	0.00	746 (1.9)	621 (1.6)	0.02
Acute Renal Failure	4321 (11.2)	75987 (11.9)	0.02	4320 (11.2)	4419 (11.5)	0.01
Chronic Kidney Disease	1048 (2.7)	13165 (2.1)	0.04	1048 (2.7)	887 (2.3)	0.03
Skin Ulcer	1379 (3.6)	26829 (4.2)	0.03	1379 (3.6)	1366 (3.5)	0.00
Severe Skin Burn or Condition	18 (0.0)	186 (0.0)	0.01	NS	NS	0.02
Head Injury	244 (0.6)	5451 (0.9)	0.03	243 (0.6)	269 (0.7)	0.01
Hip/Vertebral Fractures without Spinal Cord Injury	1821 (4.7)	35070 (5.5)	0.03	1820 (4.7)	2026 (5.3)	0.02
Limb Amputation	514 (1.3)	8013 (1.3)	0.01	513 (1.3)	513 (1.3)	0.00
Complications of Specified Implanted Device or Graft	2291 (5.9)	36222 (5.7)	0.01	2290 (5.9)	2351 (6.1)	0.01
Major Organ Transplant or Replacement Status	617 (1.6)	15676 (2.4)	0.06	617 (1.6)	687 (1.8)	0.01
Artificial Openings for Feeding or Elimination	1690 (4.4)	30867 (4.8)	0.02	1690 (4.4)	1673 (4.3)	0.00

ASD = Absolute standardized difference. NS = Not Shown due to a cell size < 11.

© 2026 Lin YL et al. *JAMA Network Open*

eTable 5. Patient demographic, clinical, and area characteristics for chemotherapy episodes before and after matching in Maryland and control states – sub sample 1 for timely receipt of presumed adjuvant chemotherapy for patients with breast, lung, and colorectal cancers

	Before match			After match		
**Patient characteristic**	**Maryland n= 2,259**	**Control states n= 35,029**	**ASD**	**Maryland n= 2,104**	**Control states n= 2,104**	**ASD**
Age at chemotherapy episode initiation, mean (SD)	71.2 (7.3)	70.8 (7.8)	0.05	71.1 (7.3)	71.2 (7.3)	0.00
Frailty index (0-1), mean (SD)	0.2 (0.0)	0.2 (0.1)	0.03	0.2 (0.0)	0.2 (0.0)	0.03
Social deprivation index of zip code (0-100), mean (SD)	41.8 (29.2)	43.0 (29.5)	0.04	41.5 (29.1)	41.2 (29.9)	0.01
% uninsured in zip code, mean (SD)	8.1 (5.2)	8.2 (5.7)	0.01	8.1 (5.2)	8.2 (5.5)	0.02
Sex, n (%)			0.07			0.02
Male	534 (23.6)	9280 (26.5)		488 (23.2)	509 (24.2)	
Female	1725 (76.4)	25749 (73.5)		1616 (76.8)	1595 (75.8)	
Race and ethnicity, n (%)			0.50			0.05
Asian/Pacific islander	59 (2.6)	554 (1.6)		57 (2.7)	58 (2.8)	
Hispanic	59 (2.6)	1367 (3.9)		57 (2.7)	62 (2.9)	
Non-Hispanic black	684 (30.3)	3695 (10.5)		552 (26.2)	507 (24.1)	
Non-Hispanic white	1432 (63.4)	28796 (82.2)		1413 (67.2)	1455 (69.2)	
Other	25 (1.1)	617 (1.8)		25 (1.2)	22 (1.0)	
Dual eligible during episode, n (%)	329 (14.6)	6532 (18.6)	0.11	308 (14.6)	307 (14.6)	0.00
Continuous Part D enrollment during episode, n (%)	1333 (59.0)	24416 (69.7)	0.22	1267 (60.2)	1242 (59.0)	0.02
Initial chemotherapy of episode			0.06			0.00
Part B	2233 (98.8)	34379 (98.1)		2096 (99.6)	2096 (99.6)	
Part D	26 (1.2)	650 (1.9)		NS	NS	
Cancer Type, n (%)			0.13			0.00
Breast cancer	1198 (53.0)	16952 (48.4)		1132 (53.8)	1132 (53.8)	
Lung Cancer	437 (19.3)	6379 (18.2)		389 (18.5)	389 (18.5)	
Colorectal cancer	624 (27.6)	11698 (33.4)		583 (27.7)	583 (27.7)	
Metastasis during episode, n (%)			0.07			0.04
Distant	419 (18.5)	7189 (20.5)		388 (18.4)	400 (19.0)	
Lymph node	229 (10.1)	3994 (11.4)		219 (10.4)	186 (8.8)	
None	1611 (71.3)	23846 (68.1)		1497 (71.2)	1518 (72.1)	
Had any nursing facility service in prior 90 days, n (%)	133 (5.9)	2502 (7.1)	0.05	112 (5.3)	120 (5.7)	0.02
Chemotherapy episode in the prior 12 months, n (%)	267 (11.8)	3930 (11.2)	0.02	251 (11.9)	242 (11.5)	0.01
Diabetes, n (%)			0.11			0.03
No diabetes	1359 (60.2)	22286 (63.6)		1302 (61.9)	1283 (61.0)	
Diabetes without complications	464 (20.5)	7404 (21.1)		424 (20.2)	417 (19.8)	
Diabetes with complication	436 (19.3)	5339 (15.2)		378 (18.0)	404 (19.2)	
Hierarchical Condition Category, n (%)						

© 2026 Lin YL et al. *JAMA Network Open*

Septicemia, Sepsis, Systemic Inflammatory Response Syndrome/Shock	160 (7.1)	2014 (5.7)	0.05	138 (6.6)	153 (7.3)	0.03
HIV/AIDS/Opportunistic Infections	31 (1.4)	378 (1.1)	0.03	28 (1.3)	27 (1.3)	0.00
Protein-Calorie Malnutrition	103 (4.6)	2392 (6.8)	0.10	98 (4.7)	103 (4.9)	0.01
Morbid Obesity	197 (8.7)	2913 (8.3)	0.01	181 (8.6)	184 (8.7)	0.01
Other Significant Endocrine and Metabolic Disorders	190 (8.4)	3150 (9.0)	0.02	175 (8.3)	183 (8.7)	0.01
End-Stage Liver Disease	35 (1.5)	552 (1.6)	0.00	29 (1.4)	27 (1.3)	0.01
Other GI diseases (Chronic Hepatitis, Chronic Pancreatitis, Inflammatory Bowel Disease)	74 (3.3)	1259 (3.6)	0.02	64 (3.0)	65 (3.1)	0.00
Intestinal Obstruction/Perforation	307 (13.6)	5608 (16.0)	0.07	281 (13.4)	270 (12.8)	0.02
Bone/Joint/ Muscle Infections /Necrosis	24 (1.1)	352 (1.0)	0.01	20 (1.0)	30 (1.4)	0.04
Rheumatoid Arthritis and Inflammatory Connective Tissue Disease	166 (7.3)	2876 (8.2)	0.03	154 (7.3)	155 (7.4)	0.00
Hematological Disorders	322 (14.3)	4751 (13.6)	0.02	294 (14.0)	303 (14.4)	0.01
Disorders of Immunity	179 (7.9)	2601 (7.4)	0.02	168 (8.0)	177 (8.4)	0.02
Drug/Alcohol Dependence	86 (3.8)	1107 (3.2)	0.04	73 (3.5)	74 (3.5)	0.00
Major psychiatric disorder	190 (8.4)	3214 (9.2)	0.03	176 (8.4)	183 (8.7)	0.01
Spinal Cord Injury/Paralytic disorders	36 (1.6)	484 (1.4)	0.02	32 (1.5)	27 (1.3)	0.02
Neurological disorders affecting motor function	77 (3.4)	1034 (3.0)	0.03	72 (3.4)	67 (3.2)	0.01
Other neurologic disorders (Multiple Sclerosis, Seizure, Coma, Brain compression / Anoxic Damage)	104 (4.6)	1459 (4.2)	0.02	91 (4.3)	82 (3.9)	0.02
Respirator Dependence/Tracheostomy Status	19 (0.8)	312 (0.9)	0.01	14 (0.7)	14 (0.7)	0.00
Cardio-respiratory failure	199 (8.8)	3131 (8.9)	0.00	175 (8.3)	184 (8.7)	0.02
Congestive Heart Failure	388 (17.2)	6557 (18.7)	0.04	361 (17.2)	371 (17.6)	0.01
Acute Myocardial Infarction / Angina / Other Acute Ischemic Heart Disease	172 (7.6)	3072 (8.8)	0.04	158 (7.5)	157 (7.5)	0.00
Specified Heart Arrhythmias	399 (17.7)	6939 (19.8)	0.06	372 (17.7)	381 (18.1)	0.01
Cerebrovascular insult	142 (6.3)	1763 (5.0)	0.05	123 (5.8)	107 (5.1)	0.03
Severe peripheral vascular disease	147 (6.5)	2102 (6.0)	0.02	131 (6.2)	115 (5.5)	0.03
Vascular Disease	489 (21.6)	8215 (23.5)	0.04	445 (21.2)	458 (21.8)	0.02
Cystic Fibrosis/Fibrosis of Lung and Other Chronic Lung Disorders	89 (3.9)	1076 (3.1)	0.05	77 (3.7)	71 (3.4)	0.02
Chronic Obstructive Pulmonary Disease	627 (27.8)	11036 (31.5)	0.08	581 (27.6)	593 (28.2)	0.01
Pneumonia	69 (3.1)	1313 (3.7)	0.04	61 (2.9)	59 (2.8)	0.01
Exudative Macular Degeneration	30 (1.3)	449 (1.3)	0.00	28 (1.3)	31 (1.5)	0.01
Acute Renal Failure	187 (8.3)	3050 (8.7)	0.02	169 (8.0)	172 (8.2)	0.01
Chronic Kidney Disease	34 (1.5)	381 (1.1)	0.04	29 (1.4)	24 (1.1)	0.02
Skin Ulcer	67 (3.0)	988 (2.8)	0.01	59 (2.8)	58 (2.8)	0.00
Severe Skin Burn or Condition	NS	NS	0.03	NS	NS	0.00
Head Injury	NS	203 (0.6)	0.03	NS	NS	0.03
Hip/Vertebral Fractures without Spinal Cord Injury	46 (2.0)	663 (1.9)	0.01	45 (2.1)	45 (2.1)	0.00
Limb Amputation	82 (3.6)	1072 (3.1)	0.03	72 (3.4)	74 (3.5)	0.01

© 2026 Lin YL et al. *JAMA Network Open*

Complications of Specified Implanted Device or Graft	100 (4.4)	1497 (4.3)	0.01	94 (4.5)	94 (4.5)	0.00
Major Organ Transplant or Replacement Status	NS	88 (0.3)	0.00	NS	NS	0.02
Artificial Openings for Feeding or Elimination	139 (6.2)	2802 (8.0)	0.07	130 (6.2)	134 (6.4)	0.01
**Hospital Service Area characteristics**						
No. of billing oncologist per 10,000 elderly population at HSA, mean (SD)	11.1 (4.4)	11.2 (11.7)	0.02	11.1 (4.4)	9.5 (9.1)	0.21
Competition (oncology practice-defined HHI at HSA), mean (SD)	0.4 (0.2)	0.5 (0.3)	0.49	0.4 (0.2)	0.5 (0.3)	0.50
No. of hospital-owned oncology practice at HSA, mean (SD)	0.4 (0.5)	1.9 (3.3)	0.60	0.4 (0.5)	1.6 (2.5)	0.63
Total no. of beds at HSA, mean (SD)	3639.4 (2744.7)	1707.2 (2372.6)	0.75	3670.9 (2737.0)	1642.6 (1926.1)	0.86
% of 340B hospitals at HSA, mean (SD)	55.3 (43.6)	67.4 (56.8)	0.24	55.2 (42.9)	61.3 (50.0)	0.13
% of teaching hospitals at HSA, mean (SD)	64.5 (36.8)	63.2 (40.5)	0.03	65.0 (36.5)	67.2 (35.8)	0.06

ASD = Absolute standardized difference. NS = Not Shown due to a cell size < 11.

© 2026 Lin YL et al. *JAMA Network Open*

eTable 6. Patient demographic, clinical, and area characteristics for chemotherapy episodes before and after matching in Maryland and control states – sub sample 2 for end-of-life measures

	Before match			After match		
**Patient characteristic**	**Maryland n= 8,312**	**Control states n= 139,470**	**ASD**	**Maryland n= 8,281**	**Control states n= 8,281**	**ASD**
Age at chemotherapy episode initiation, mean (SD)	74.3 (8.6)	74.1 (8.9)	0.02	74.3 (8.6)	74.4 (8.9)	0.01
Frailty index (0-1), mean (SD)	0.2 (0.1)	0.2 (0.1)	0.08	0.2 (0.1)	0.2 (0.1)	0.00
Social deprivation index of zip code (0-100), mean (SD)	41.0 (29.4)	42.0 (29.3)	0.04	41.0 (29.4)	40.6 (29.6)	0.01
% uninsured in zip code, mean (SD)	7.9 (5.2)	7.9 (5.7)	0.00	7.9 (5.2)	8.0 (5.4)	0.02
Sex, n (%)			0.02			0.01
Male	4100 (49.3)	70445 (50.5)		4085 (49.3)	4146 (50.1)	
Female	4212 (50.7)	69025 (49.5)		4196 (50.7)	4135 (49.9)	
Race and ethnicity, n (%)			0.43			0.09
Asian/Pacific islander	242 (2.9)	2304 (1.7)		242 (2.9)	269 (3.2)	
Hispanic	150 (1.8)	4633 (3.3)		150 (1.8)	164 (2.0)	
Non-Hispanic black	2000 (24.1)	12199 (8.7)		1970 (23.8)	1826 (22.1)	
Non-Hispanic white	5806 (69.9)	118066 (84.7)		5805 (70.1)	5910 (71.4)	
Other	114 (1.4)	2268 (1.6)		114 (1.4)	112 (1.4)	
Dual eligible during episode, n (%)	1147 (13.8)	26203 (18.8)	0.14	1144 (13.8)	1212 (14.6)	0.02
Continuous Part D enrollment during episode, n (%)	5270 (63.4)	99695 (71.5)	0.17	5247 (63.4)	5188 (62.6)	0.01
Initial chemotherapy of episode			0.07			0.00
Part B	7516 (90.4)	123287 (88.4)		7497 (90.5)	7497 (90.5)	
Part D	796 (9.6)	16183 (11.6)		784 (9.5)	784 (9.5)	
Cancer type, n (%)			0.21			0.09
Acute leukemia	173 (2.1)	2617 (1.9)		172 (2.1)	163 (2.0)	
Bladder cancer	293 (3.5)	4745 (3.4)		293 (3.5)	298 (3.6)	
Breast cancer	682 (8.2)	10011 (7.2)		675 (8.2)	665 (8.0)	
Chronic leukemia	176 (2.1)	2874 (2.1)		175 (2.1)	168 (2.0)	
Endocrine tumor	85 (1.0)	1501 (1.1)		85 (1.0)	83 (1.0)	
Female genitourinary cancer	185 (2.2)	2984 (2.1)		185 (2.2)	188 (2.3)	
Gastro/esophageal cancer	335 (4.0)	6093 (4.4)		334 (4.0)	331 (4.0)	
Head and Neck Cancer	224 (2.7)	3821 (2.7)		224 (2.7)	223 (2.7)	
Kidney cancer	99 (1.2)	1857 (1.3)		95 (1.1)	86 (1.0)	
Liver cancer	182 (2.2)	3878 (2.8)		181 (2.2)	174 (2.1)	
Lung cancer	2208 (26.6)	35445 (25.4)		2202 (26.6)	2261 (27.3)	
Lymphoma	461 (5.5)	8067 (5.8)		461 (5.6)	433 (5.2)	
Myelodysplastic syndromes	240 (2.9)	3921 (2.8)		239 (2.9)	237 (2.9)	
Multiple myeloma	411 (4.9)	6004 (4.3)		409 (4.9)	407 (4.9)	
Ovarian cancer	300 (3.6)	4309 (3.1)		299 (3.6)	289 (3.5)	
Pancreatic cancer	650 (7.8)	9891 (7.1)		647 (7.8)	624 (7.5)	
Prostate cancer	257 (3.1)	4107 (2.9)		255 (3.1)	276 (3.3)	
Metastasis to other and unspecified sites	137 (1.6)	4033 (2.9)		137 (1.7)	161 (1.9)	
Metastasis to respiratory and	56 (0.7)	3023 (2.2)		55 (0.7)	53 (0.6)	

© 2026 Lin YL et al. *JAMA Network Open*

digestive organs						
Colorectal and small intestine cancer	609 (7.3)	10709 (7.7)		609 (7.4)	640 (7.7)	
Other cancer	549 (6.6)	9580 (6.9)		549 (6.6)	521 (6.3)	
Metastasis during episode, n (%)			0.06			0.02
Distant	5403 (65.0)	94335 (67.6)		5380 (65.0)	5425 (65.5)	
Lymph node	189 (2.3)	3420 (2.5)		188 (2.3)	178 (2.1)	
None	2720 (32.7)	41715 (29.9)		2713 (32.8)	2678 (32.3)	
Had any nursing facility service in prior 90 days, n (%)	529 (6.4)	11508 (8.3)	0.07	527 (6.4)	543 (6.6)	0.01
Chemotherapy episode in the prior 12 months, n (%)	4110 (49.4)	70668 (50.7)	0.02	4095 (49.5)	4107 (49.6)	0.00
Diabetes, n (%)			0.03			0.03
No diabetes	5139 (61.8)	86277 (61.9)		5121 (61.8)	5214 (63.0)	
Diabetes without complications	1541 (18.5)	27612 (19.8)		1537 (18.6)	1515 (18.3)	
Diabetes with complication	1632 (19.6)	25581 (18.3)		1623 (19.6)	1552 (18.7)	
Hierarchical Condition Category, n (%)						
Septicemia, Sepsis, Systemic Inflammatory Response Syndrome/Shock	978 (11.8)	16734 (12.0)	0.01	974 (11.8)	960 (11.6)	0.01
HIV/AIDS/Opportunistic Infections	224 (2.7)	3348 (2.4)	0.02	222 (2.7)	217 (2.6)	0.00
Protein-Calorie Malnutrition	798 (9.6)	18506 (13.3)	0.12	796 (9.6)	784 (9.5)	0.00
Morbid Obesity	366 (4.4)	6755 (4.8)	0.02	364 (4.4)	337 (4.1)	0.02
Other Significant Endocrine and Metabolic Disorders	1141 (13.7)	21978 (15.8)	0.06	1137 (13.7)	1159 (14.0)	0.01
End-Stage Liver Disease	251 (3.0)	5312 (3.8)	0.04	250 (3.0)	232 (2.8)	0.01
Other GI diseases (Chronic Hepatitis, Chronic Pancreatitis, Inflammatory Bowel Disease)	345 (4.2)	5507 (3.9)	0.01	341 (4.1)	347 (4.2)	0.00
Intestinal Obstruction/Perforation	706 (8.5)	11559 (8.3)	0.01	704 (8.5)	720 (8.7)	0.01
Bone/Joint/Muscle Infections/Necrosis	177 (2.1)	3336 (2.4)	0.02	176 (2.1)	179 (2.2)	0.00
Rheumatoid Arthritis and Inflammatory Connective Tissue Disease	546 (6.6)	11152 (8.0)	0.05	544 (6.6)	537 (6.5)	0.00
Hematological Disorders	2331 (28.0)	41978 (30.1)	0.05	2321 (28.0)	2276 (27.5)	0.01
Disorders of Immunity	2003 (24.1)	37748 (27.1)	0.07	1997 (24.1)	2011 (24.3)	0.00
Drug/Alcohol Dependence	340 (4.1)	5527 (4.0)	0.01	337 (4.1)	363 (4.4)	0.02
Major psychiatric disorder	609 (7.3)	11203 (8.0)	0.03	606 (7.3)	604 (7.3)	0.00
Spinal Cord Injury/Paralytic disorders	301 (3.6)	5215 (3.7)	0.01	301 (3.6)	311 (3.8)	0.01
Neurological disorders affecting motor function	463 (5.6)	8938 (6.4)	0.04	463 (5.6)	454 (5.5)	0.00
Other neurologic disorders (Multiple Sclerosis, Seizure, Coma, Brain Compression/Anoxic Damage)	619 (7.4)	10761 (7.7)	0.01	614 (7.4)	636 (7.7)	0.01
Respirator Dependence/Tracheostomy Status	103 (1.2)	1968 (1.4)	0.02	102 (1.2)	101 (1.2)	0.00
Cardio-respiratory failure	882 (10.6)	17556 (12.6)	0.06	881 (10.6)	848 (10.2)	0.01
Congestive Heart Failure	2143 (25.8)	38099 (27.3)	0.03	2135 (25.8)	2102 (25.4)	0.01
Acute Myocardial Infarction/Angina/Other Acute Ischemic Heart Disease	718 (8.6)	15316 (11.0)	0.08	716 (8.6)	708 (8.5)	0.00
Specified Heart Arrhythmias	2036 (24.5)	36998 (26.5)	0.05	2031 (24.5)	1999 (24.1)	0.01
Cerebrovascular insult	810 (9.7)	12045 (8.6)	0.04	805 (9.7)	806 (9.7)	0.00

© 2026 Lin YL et al. *JAMA Network Open*

Severe peripheral vascular disease	958 (11.5)	15365 (11.0)	0.02	954 (11.5)	921 (11.1)	0.01
Vascular Disease	2447 (29.4)	44053 (31.6)	0.05	2438 (29.4)	2425 (29.3)	0.00
Cystic Fibrosis/Fibrosis of Lung and Other Chronic Lung Disorders	383 (4.6)	5949 (4.3)	0.02	381 (4.6)	405 (4.9)	0.01
Chronic Obstructive Pulmonary Disease	2884 (34.7)	54263 (38.9)	0.09	2876 (34.7)	2848 (34.4)	0.01
Pneumonia	529 (6.4)	10383 (7.4)	0.04	529 (6.4)	546 (6.6)	0.01
Exudative Macular Degeneration	183 (2.2)	2949 (2.1)	0.01	182 (2.2)	186 (2.2)	0.00
Acute Renal Failure	1383 (16.6)	24242 (17.4)	0.02	1379 (16.7)	1387 (16.7)	0.00
Chronic Kidney Disease	240 (2.9)	3025 (2.2)	0.05	239 (2.9)	216 (2.6)	0.02
Skin Ulcer	378 (4.5)	7578 (5.4)	0.04	377 (4.6)	356 (4.3)	0.01
Severe Skin Burn or Condition	NS	46 (0.0)	0.02	NS	NS	0.01
Head Injury	79 (1.0)	1643 (1.2)	0.02	79 (1.0)	88 (1.1)	0.01
Hip/Vertebral Fractures without Spinal Cord Injury	467 (5.6)	9423 (6.8)	0.05	463 (5.6)	496 (6.0)	0.02
Limb Amputation	150 (1.8)	2197 (1.6)	0.02	149 (1.8)	116 (1.4)	0.03
Complications of Specified Implanted Device or Graft	653 (7.9)	10544 (7.6)	0.01	651 (7.9)	634 (7.7)	0.01
Major Organ Transplant or Replacement Status	104 (1.3)	2351 (1.7)	0.04	103 (1.2)	93 (1.1)	0.01
Artificial Openings for Feeding or Elimination	541 (6.5)	9904 (7.1)	0.02	541 (6.5)	542 (6.5)	0.00
**HSA Characteristics**						
No. of billing oncologist per 10,000 elderly population at HSA, mean (SD)	11.3 (4.2)	12.1 (12.4)	0.09	11.3 (4.2)	10.0 (9.5)	0.18
Competition (oncology practice-defined HHI at HSA), mean (SD)	0.4 (0.2)	0.5 (0.3)	0.52	0.4 (0.2)	0.5 (0.3)	0.56
No. of hospital-owned oncology practice at HSA, mean (SD)	0.5 (0.5)	2.1 (3.6)	0.63	0.5 (0.5)	1.6 (2.3)	0.68
Total no. of beds at HSA, mean (SD)	3948.6 (2655.5)	1864.6 (2493.9)	0.81	3951.8 (2654.6)	1697.2 (1800.5)	0.99
% of 340B hospitals at HSA, mean (SD)	57.7 (40.4)	67.4 (56.3)	0.20	57.6 (40.4)	60.2 (47.4)	0.06
% of teaching hospitals at HSA, mean (SD)	68.0 (33.9)	65.0 (39.9)	0.08	68.0 (33.9)	67.8 (34.6)	0.01

ASD = Absolute standardized difference. NS = Not shown due to a cell count < 11.

© 2026 Lin YL et al. *JAMA Network Open*

eAppendix 7. Sensitivity Analyses

We performed sensitivity analyses by excluding the year of 2014 from the post period. The results are summarized in eTable 7.

© 2026 Lin YL et al. *JAMA Network Open*

eTable 7. Adjusted means for each outcome measure and Difference-in-Differences estimates

Outcome measure	Adjusted means (SDs), $ or %[a]				DID estimates[a]		
	Pre-GBR implementation (2011-2013)		Post-GBR implementation (2015-2018)				
	Maryland	Control states	Maryland	Control states	DID (95% CI), $ or percentage points	*P* value	FDR adjusted *P* value[b]
**Payments**							
Total episode payments	$48345 ($513)	$45899 ($659)	$51518 ($2672)	$52693 ($2974)	-$3766 (-$5099, -$2394)	< 0.001	< 0.001
Total episode hospital payments (inpatient and outpatient)	$15123 ($505)	$20924 ($448)	$14857 ($110)	$24344 ($703)	-$4010 (-$4931, -$3027)	< 0.001	< 0.001
Total episode professional payments	$14958 ($774)	$11654 ($692)	$15847 ($1385)	$11605 ($996)	$1820 ($1113, $2566)	< 0.001	< 0.001
**Hospital-based utilization**							
All-cause hospitalization	39.8 (1.2)	39.0 (1.8)	34.7 (1.1)	35.5 (1.2)	-1.6 (-3.3, 0.1)	0.06	0.12
All-cause ED visit	11.7 (0.6)	13.2 (1.1)	13.6 (0.6)	13.3 (0.1)	1.4 (-0.1, 2.8)	0.07	0.12
**Quality of care**							
Chemotherapy-related hospitalization	16.3 (0.9)	14.7 (1.5)	14.2 (0.4)	14.2 (0.4)	-1.6 (-3.1, -0.2)	0.02	0.07
Chemotherapy-related ED visit	6.0 (0.2)	6.1 (0.1)	6.0 (0.2)	6.2 (0.3)	-0.2 (-1.1, 0.8)	0.77	0.93
Timely receipt of chemotherapy	63.4 (1.0)	68.4 (2.2)	67.6 (4.1)	68.2 (3.2)	7.7 (-0.4, 15.7)	0.06	0.12
No or late hospice enrollment[c]	48.8 (2.5)	46.7 (2.2)	46.8 (1.6)	47.7 (2.0)	0.3 (-4.1, 4.6)	0.91	0.93
>1 ED visit in last 30 d of life	16.1 (1.2)	13.7 (1.3)	16.5 (1.5)	17.0 (1.7)	-1.5 (-4.8, 1.7)	0.35	0.53
ICU stay in last 30 d of life	22.0 (2.1)	24.1 (1.2)	23.0 (1.5)	25.9 (0.9)	-0.4 (-4.0, 3.3)	0.85	0.93
Receipt of chemotherapy in last 14 d of life	11.8 (0.5)	10.1 (0.7)	10.8 (1.2)	10.6 (0.3)	-0.1 (-2.9, 2.6)	0.93	0.93

Abbreviations: DID, difference-in-differences; ED, emergency department; FDR, false 1 discovery rate; GBR, Global Budget Revenue; ICU, intensive care unit.

[a]All models were adjusted for patient age, sex, race and ethnicity, dual eligibility, institutional status, disability index, Part D enrollment, part B or D chemotherapy, cancer type, metastasis status, any prior chemotherapy episode, comorbidity using Hierarchical Condition 6 Categories groups, zip code level Social Deprivation Index and percentage of uninsured people, and year-specific Hospital Service Area (HSA) level variables (number of billing oncologists per 10 000 elderly population, oncology practice defined Herfindahl-Hirschman index, number of hospital-owned oncology practices, total number of beds, percentage of 340B hospitals, and percentage of teaching hospitals). Year (based on chemotherapy initiation) fixed effects were also included. DiDs calculated directly from the adjusted means (presented in the first 4 columns) are slightly different from the DiD estimates (presented in

© 2026 Lin YL et al. *JAMA Network Open*

the final column) because the regression models that generated the adjusted rates for the former estimates did not include HSA fixed effects, while the latter estimates included HSA fixed effects.
[b]This adjustment approach is also known as Benjamini-Hochberg procedure. We investigated 12 outcome variables and used the Benjamini-Hochberg procedure to adjust these 12 P values.
[c]Late hospice enrollment is defined as hospice enrollment within 3 days of death.

© 2026 Lin YL et al. *JAMA Network Open*

eReferences

1. The Center for Medicare and Medicaid Innovation. Oncology Care Model Performance Period 3 and Forward Payment Methodology. Updated November 12, 2024. Accessed March 20, 2025. https://innovation.cms.gov/innovation-models/oncology-care
2. Kakani P. The impact of vertical integration on health care delivery and costs: Evidence from physician-pharmacy integration. *J Health Econ*. Dec 2025;104:103085. doi:10.1016/j.jhealeco.2025.103085
3. Acumen. *Maryland Model Analytics Consultant Report on Evaluation of Maryland Medicare Spending on Inpatient Care*. 2022. Accessed June 20, 2024. https://www.crisphealth.org/learning-system/md-model-analytics/
4. Acumen. *Maryland Model Analytics Consultant Report on Evaluation of Maryland Medicare Spending on Inpatient Care*. November 2022. Accessed June 20, 2024. https://www.crisphealth.org/learning-system/md-model-analytics/
5. Centers for Medicare & Medicaid Services. *CMS Standardization Methodology For Allowed Amount– v.13.* 2024. Accessed August 23, 2024. https://resdac.org/articles/cms-payment-standardization-overview
6. Keating NL, Jhatakia S, Brooks GA, et al. Association of Participation in the Oncology Care Model With Medicare Payments, Utilization, Care Delivery, and Quality Outcomes. *JAMA*. Nov 9 2021;326(18):1829-1839. doi:10.1001/jama.2021.17642
7. Jalali A, Martin C, Nelson RE, et al. Provider Practice Competition and Adoption of Medicare's Oncology Care Model. *Med Care*. Feb 2020;58(2):154-160. doi:10.1097/mlr.0000000000001243
8. Bilinski A, Hatfield LA. Nothing to see here? Non-inferiority approaches to parallel trends and other model assumptions. 2018:arXiv:1805.03273. doi:10.48550/arXiv.1805.03273 https://ui.adsabs.harvard.edu/abs/2018arXiv180503273B
9. Schmitt M. Do hospital mergers reduce costs? *J Health Econ*. Mar 2017;52:74-94. doi:10.1016/j.jhealeco.2017.01.007

© 2026 Lin YL et al. *JAMA Network Open*
