# Supplementary material for: Global Budget Revenue Model and Care for Patients Receiving Chemotherapy
Source: JAMA Netw Open. 2026 Mar 5;9(3):e260485. doi: 10.1001/jamanetworkopen.2026.0485 (PMC12964157; doi:10.1001/jamanetworkopen.2026.0485)
Supplement: Supplement 2. — Data Sharing Statement [file jamanetwopen-e260485-s002.pdf]

Data Sharing Statement

Lin. Global Budget Revenue Model and Care for Patients Receiving Chemotherapy. *JAMA Netw Open*. Published March 05, 2026. doi:10.1001/jamanetworkopen.2026.0485

Data
Data available: No
